# Optimized sequencing depth and de novo assembler for deeply reconstructing the transcriptome of the tea plant, an economically important plant species

**DOI:** 10.1186/s12859-019-3166-x

**Published:** 2019-11-06

**Authors:** Fang-Dong Li, Wei Tong, En-Hua Xia, Chao-Ling Wei

**Affiliations:** 1 State Key Laboratory of Tea Plant Biology and Utilization, Anhui Agricultural University, Hefei, 230036 China; 2 School of Science, Anhui Agricultural University, Hefei, 230036 China

**Keywords:** Tea plant, *Camellia sinensis*, Transcriptome, de novo assembly, Sequencing depth

## Abstract

**Background:**

Tea is the oldest and among the world’s most popular non-alcoholic beverages, which has important economic, health and cultural values. Tea is commonly produced from the leaves of tea plants (*Camellia sinensis*), which belong to the genus *Camellia* of family *Theaceae*. In the last decade, many studies have generated the transcriptomes of tea plants at different developmental stages or under abiotic and/or biotic stresses to investigate the genetic basis of secondary metabolites that determine tea quality. However, these results exhibited large differences, particularly in the total number of reconstructed transcripts and the quality of the assembled transcriptomes. These differences largely result from limited knowledge regarding the optimized sequencing depth and assembler for transcriptome assembly of structurally complex plant species genomes.

**Results:**

We employed different amounts of RNA-sequencing data, ranging from 4 to 84 Gb, to assemble the tea plant transcriptome using five well-known and representative transcript assemblers. Although the total number of assembled transcripts increased with increasing sequencing data, the proportion of unassembled transcripts became saturated as revealed by plant BUSCO datasets. Among the five representative assemblers, the Bridger package shows the best performance in both assembly completeness and accuracy as evaluated by the BUSCO datasets and genome alignment. In addition, we showed that Bridger and BinPacker harbored the shortest runtimes followed by SOAP*denovo* and Trans-ABySS.

**Conclusions:**

The present study compares the performance of five representative transcript assemblers and investigates the key factors that affect the assembly quality of the transcriptome of the tea plants. This study will be of significance in helping the tea research community obtain better sequencing and assembly of tea plant transcriptomes under conditions of interest and may thus help to answer major biological questions currently facing the tea industry.

## Background

The power of RNA sequencing (RNA-seq) is the fact that the twin aspects of discovery and quantification can be combined in a single high-throughput RNA sequencing experiment. With the rapid innovation of sequencing technology, the scale of raw data generation is growing explosively [1]. Increasing the number of reads should increase the level of uniquely mapped reads to facilitate the de novo assembly and quantification analysis, but this approach can also increase the cost of library preparation and sequencing [2], which can be wasteful in terms of data storage and processing. Thus, the determination of a proper sequencing depth is important across all experiments, including RNA-seq, genome re-sequencing and de novo sequencing. In RNA-seq, transcript identification is accepted as playing an important role in gene discovery, as it is a key intermediate bridge between the genome and proteome. The assembly and annotation of transcripts, especially novel transcripts or alternatively spliced transcripts, is of great importance in RNA-seq analysis [3].

For an organism that lacks a high-quality reference genome, the de novo assembly of its transcriptome is a useful method for researchers to find the transcripts or genes responsive to various treatments and to better understand the expression patterns of the candidate genes. However, two major problems in de novo assembly have obstructed progress in high-throughput transcript identification: 1) most of the transcripts assembled are not full-length, and 2) only a certain proportion of the genes can be assembled. Some genes only show a high expression abundance under specific conditions or treatments, which means that multiple organs or time-series sampling need to be performed to obtain more transcripts. In addition, the de novo assembled transcripts using the default parameters in most current assemblers are usually short in length, which hampers further sequence-based analysis and experiments. Therefore, a well-assembled, accurate, and comprehensive transcriptome is a prerequisite for the subsequent analysis. The accurate assembly of a transcript is largely determined by the sequencing depth, which leads to the question of how much data should be generated in an RNA-seq experiment to obtain robust results. Some recent reports suggest that approximately 700 million reads are required to obtain accurate quantification of > 95% of expressed transcripts in a mammalian genome [4], but there are no systematic analyses illustrating the effect of sequencing depth on transcript assembly [5].

Currently, many programs or tools [6–10] have been developed for the assembly of transcriptomic data. Trinity has been widely adopted in many experiments due to its considerably improved assembly performance with an exhaustive enumeration algorithm to search for isoform-representing paths in a *de Bruijn* graph; this type of analysis makes the algorithm highly sensitive to splicing isoforms but suffers from high false positives. Bridger aims to build a bridge between the key ideas of two popular assemblers, the reference-based assembler Cufflinks [11] and the de novo assembler Trinity [6], which specifically generalized the main techniques employed by Cufflinks to overcome the limitations of Trinity. BinPacker assembles full-length transcripts by remodeling the problem of tracking a set of trajectories of items over a splicing graph, which is constructed by employing the techniques used in Bridger with several updates [10].

Tea is the oldest and among the most popular nonalcoholic beverage in the world [12, 13]. Tea is commonly produced from the young leaves of tea plants, which are widely grown in more than 52 countries. Currently, tea has increasingly become the major source of income in tea-producing countries [14]. In the last two years, two draft genomes of tea plants (*C. sinensis* var*. sinensis* and *C. sinensis* var*. assamica*) have been released [15, 16]. Both genomes possessed a giant nuclear genome size of ~ 3 Gb. With the rapid development of RNA-seq, many transcriptomic data have been generated in tea plants across different developmental stages, various biotic and abiotic stresses [14, 17–30]. However, due to the absence of a previously constructed high-quality reference genome and the complexities of the tea plant genome, it is difficult to de novo assemble a good transcript sets or to identify novel transcripts, even with reference-based analyses. In tea plants, some transcriptomic studies only focused on only one or few tissues [18, 31–40], whereas others did multiple samples [41–49] or even with pooled samples [17, 37, 50], making it difficult to assemble transcripts in standard. The amount of data generated among different studies also varies from 1 Gb to 85 Gb and most of the studies only use a single tool (mostly Trinity) for transcript assembly (Additional file 1: Table S1).

This abundance of transcriptomic studies encourages us to perform a performance evaluation of different assemblers in tea plants. We also assessed the effect of different sequencing depths on the transcriptome assembly of tea plants and attempted to find an optimized data size that performs better in a range extending from 4 Gb/1 Gb to 84 Gb/11 Gb for pooled/single samples. We neither aim to provide an exhaustive compilation of resources or software tools nor to indicate the best analysis pipeline, but rather to provide an annotated guideline for RNA-seq data analysis in tea plants and other similar plant species.

## Results

### RNA sequencing and de novo assembly using five representative assemblers

To investigate the suitable de novo assembler and preferred sequencing depth for tea plant transcriptome assembly, we previously sequenced the transcriptome of tea plants derived from eight characteristic tissues (apical bud, first young leaf, second young leaf, mature leaf in summer, stem, flower, fruit, and root) using an Illumina Hiseq2000 platform [51]. This approach yielded a total of 94.1 Gb of raw RNA-seq data with at least 131 million reads per sample. The average GC content was 46.10% (Additional file 1: Table S2).

**Fig. 1:**
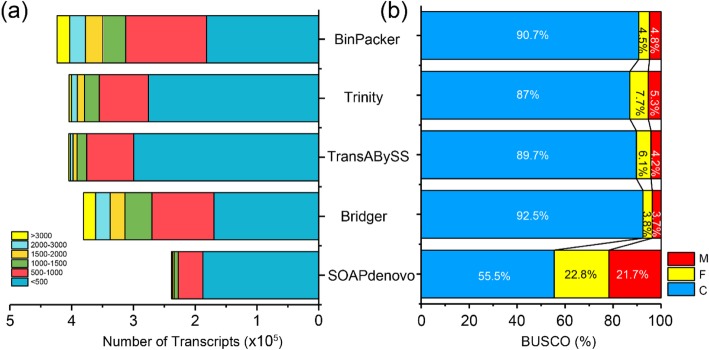
Overview of the transcriptome assemblies using five state-of-the-art assemblers. **a** Length distributions. **b** BUSCO completeness assessment. M: Missing BUSCOs; F: Fragmented BUSCOs; C: Complete BUSCOs

We assembled the RNA-seq data using five popular and typical de novo assemblers, including Trinity, Trans-ABySS, SOAP*denovo*, BinPacker and Bridger. The parameters used for the analyses were default with the exception of *k*-mer values, which ranged from 19 to 32. All assemblies were run on a single-node machine with 1024 GB memory and 8 Intel 12-core processors. The results show that, among the five assemblers, BinPacker harvested the largest number of transcripts (423,768) followed by Trans-ABySS and Trinity assembly, which was approximately 1.8-fold greater than the SOAP*denovo* assembly (Table 1). The assembled transcripts also varied in length. Bridger assembly exhibited the largest maximum, average and N50 lengths; these were similar in length to those of BinPacker assembly but were considerably larger than those of the Trans-ABySS and Trinity assemblies. The overall length distribution shows that, compared to Trans-ABySS and Trinity assembly, BinPacker and Bridger assemblies harbored the smallest proportions of transcripts shorter than 500 bp, and over 25% of the assembled transcripts were longer than 1 kb (Fig. 1a). We further investigated the shared and specific transcripts assembled from five assemblers. The results show that all five assemblers host a core set of 61,836 transcripts, and only an average proportion of 23.4% is assembler specific. (Additional file 1: Figure S1).

**Table 1:** Statistics of the transcriptome assemblies using five representative assemblers with 32 Gb sequencing data

Assembler	No. transcript	Total length (bp)	Maximum transcript (bp)	Average length (bp)	N50 (bp)
BinPacker	423,768	368,682,397	21,031	870.01	1348
Bridger	380,605	364,039,338	22,041	956.48	1539
SOAP*denovo*	238,364	99,912,984	14,281	419.16	443
Trans-ABySS	404,455	191,731,319	17,014	474.05	527
Trinity	404,125	225,905,337	14,565	559	701

We adopt the BUSCO (Benchmarking Universal Single-Copy Orthologs) datasets from plant lineage to assess the completeness of transcriptome assembly. Of the 1440 expected plant BUSCO orthologs, the Bridger assembly identified 92.5 and 3.8% as complete and fragmented, respectively; only 3.7% were considered missing (Fig. 1b). These results were comparable to those of the BinPacker assembly and were slightly better than the results obtained using Trans-ABySS and Trinity assemblers, which indicated that Bridger yielded a more complete transcriptome assembly.

### Evaluating coverage and integrity of assembled transcripts using genomic data

The release of the tea plant genome [15] has provided a reference to assess the accuracy and integrity of transcripts assembled from different assemblers. We first aligned the assembled transcripts to tea plant genomic sequences using BLAT to evaluate the performance of each assembler. The result show that an average of ~ 98.4% of the transcripts from five assemblers are mapped (coverage > 50%), indicating high mapping rates for the transcriptome assembly (Additional file 1: Table S3).

**Fig. 2:**
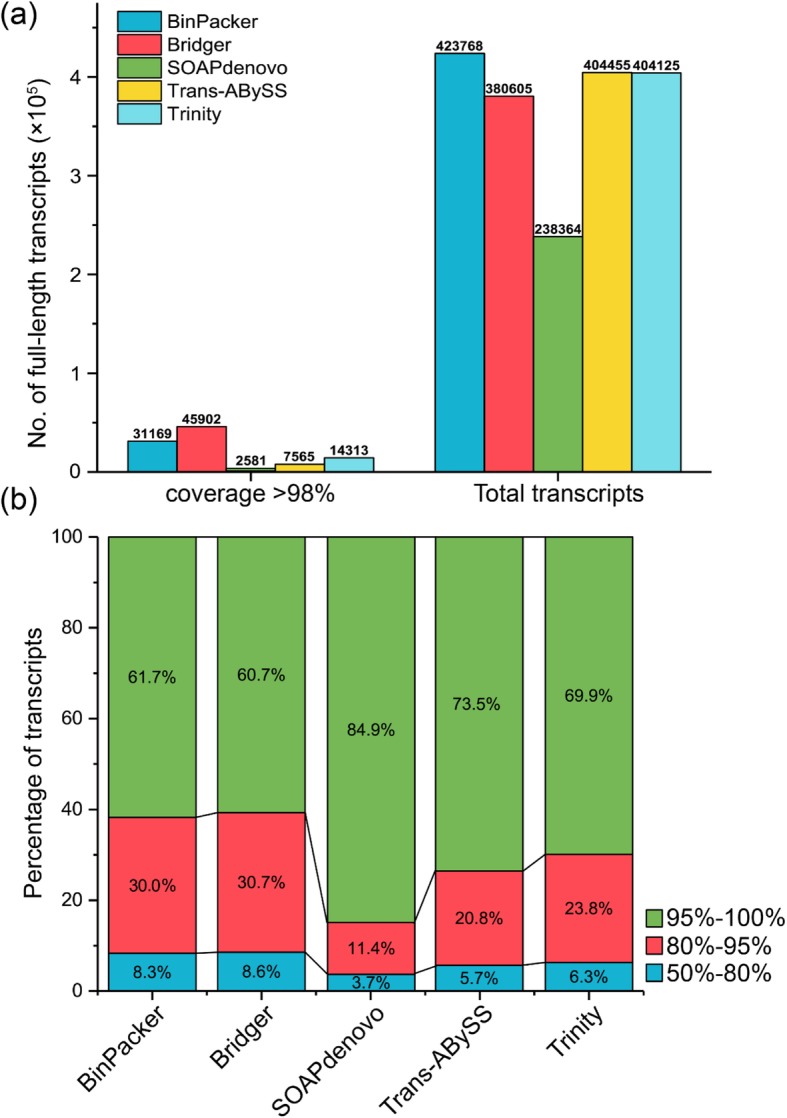
Quality evaluation of transcriptome assemblies using genome alignment. **a** The total number of constructed full-length transcripts. **b** Percentage of the transcripts with sequence identity ≥50%

We further investigated the assembly quality of transcripts at the gene level by aligning the transcripts from different assemblers to the coding sequences (CDS) of the tea plant genome and examined the numbers of reconstructed full-length (FL) genes. We found that the total number of FL transcripts (coverage ≥98%) reconstructed by different assemblers varied significantly, and ranged from 2581 (SOAP*denovo*; 1.08%) to 45,902 (Bridger; 12.06%) with an average of 20,306. Approximately 7.36% (31,169) of the transcripts from BinPacker assembly are defined as FL (Fig. 2a). This proportion is less than that of Bridger but much larger than those of the other three assemblers. Additionally, we assessed the accuracy of each reconstructed FL transcript under three levels, including identity between 50-80%, 80–95%, and over 95%. The results indicate that, of the assemblies from five assemblers, Bridger assembly reconstructed the largest number of FL transcripts (27,641) with high accuracy (identity ≥95%), followed by BinPacker assembly (19,090), Trinity assembly (9928), Trans-ABySS assembly (5533), and SOAP*denovo* assembly (2182) (Fig. 2b). We also examined the mapping rate of assembled transcripts with different coverage and identity thresholds (Additional file 1: Figure S2). This analysis indicates a good performance of Bridger and BinPacker for the construction of full-length transcripts in tea plants.

### Assessing the sensitivity of assemblers to genes expressed at different levels

**Fig. 3:**
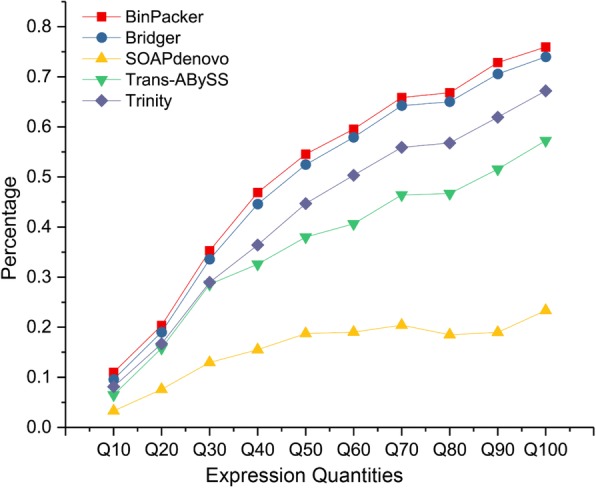
Full-length genes reconstructed by each assembler at different expression quintiles

It is a considerable challenge to reconstruct the lowly expressed gene using most current assemblers because these genes are more likely to be fragmented and/or have assembly errors. To investigate the assembly capabilities of current assemblers for genes expressed at different levels, we separately classified genes assembled from different assemblers into ten equal groups according to their expression levels from low to high and then examined their proportions that can be fully and correctly rebuilt. The results show that, as expected, the proportion of fully recovered genes increased at higher expression levels. About 60% (from 23 to 76%) of the fully recovered genes come from the top 10% of highly expressed transcripts (Fig. 3). Although all five assemblers exhibited a poor performance for low expression gene reconstruction, the BinPacker and Bridger assemblers still possessed a high amount of full-length transcripts (Fig. 2a). All five assemblers performed well for highly expressed transcripts (up 50% quantiles) with an average construction rate of more than 52%. In particular, Bridger assembly harvests the largest amount of full-length transcripts compared to the other four assemblies on average, indicating that Bridger is highly capable of tea plant transcriptome assembly. We evaluated the assembly performance for lowly expressed (FPKM < 0.1) transcripts according to the suggestions from previous study [52]. We found that only a small proportion of lowly expressed transcripts assembled from Bridger (11.1%), BinPacker (12.5%), SOAP*denovo* (6.7%), Trans-ABySS (10.3%), and Trinity (13.3%) supported by evidence of: 1) read depth > 2; 2) genome mapping rate > 50%; 3) CDS mapping rate > 60%; and 4) NR annotations (Additional file 1: Figure S3). This finding suggests the limitations of the current de novo transcriptome assemblers on the lowly expressed transcripts of tea plants, which is similar to those found in other plant species [53–55]. Nevertheless, the assembly accuracy of BinPacker, Bridger and Trinity are comparable and much higher than that of SOAP*denovo* and Trans-ABySS regarding the construction of lowly expressed transcripts. In addition to lowly expressed transcripts, we also investigated the supported evidence of sequence alignment and NR annotation of transcripts with expression levels ≥0.1. These transcripts were divided into three groups: 1) 0.1 ≤ FPKM < 1, 2) 1 ≤ FPKM < 5, and 3) FPKM ≥5. The results show that the quality of the assembled transcripts increased with increasing expression levels (Additional file 1: Figure S3). Compared to the other three assemblers, Bridger and BinPacker showed better performance of transcript assembly not only for weakly but also highly expressed transcripts. Furthermore, we examined the completeness and integrity of the assembled transcripts at different expression levels. We found that highly expressed transcripts exhibit higher assembly completeness and integrity than weakly expressed transcripts (Additional file 1: Figure S4). It is not surprising that weakly expressed transcripts are always truncated and shorted sequences that usually lack sufficient sequencing reads and evidence during transcriptome reconstruction.

### Selection of *k*-mer value

*De Bruijn* graph is the most commonly used algorithm among the current RNA-seq assemblers, of which *k*-mer is an important parameter that influences the assembly accuracy and efficiency. According to the assessment we conducted, Bridger shows the best performance for tea plant transcriptome assembly. To examine the most suitable *k*-mer values for this assembler, we further investigated the assembly quality by changing *k*-mer values from 19 to 32 (Table 2). The results showed an increase in the total number of assembled transcripts and decreases in both maximum sequence length and N50 with higher *k*-mer values (Additional file 1: Table S4). The results from *k* = 21 yielded the longest transcript and maximum N50 size, but it had a high miss rate compared to results from other *k*-mer values. The assemblies from *k* = 25 and 27 exhibit a comparable assembly completeness with the lowest missing rate of 3.7% among all *k*-mer values; however, the results from *k* = 25 harvests the longest transcript length and N50 size, which are better than the assembly from 27 *k*-mers (Table 2). This outcome indicates that a *k*-mer value of 25 is the most suitable for tea plant transcriptome assembly using Bridger.

**Table 2:** Summary of the Bridger assemblies using different *k-*mer values

*k*-mer	No. transcripts	Maximum length (bp)	Average length (bp)	N50 (bp)	BUSCO^a^		
					C	F	M
19	273,893	27,833	1003.51	1531	56.10%	22.30%	21.60%
21	350,330	28,766	987.98	1554	86.70%	6.70%	6.60%
23	370,677	18,579	948.81	1524	91.20%	4.40%	4.40%
25	380,605	22,041	956.48	1539	92.50%	3.80%	3.70%
27	383,138	20,166	951.48	1524	93.00%	3.30%	3.70%
29	383,689	19,347	950.33	1513	86.70%	6.70%	6.60%
32	390,761	19,996	896.6	1430	92.70%	3.30%	4.00%

^a^*M* Missing BUSCOs, *F* Fragmented BUSCOs, *C* Complete BUSCOs

### Optimal data architecture for de novo assembly of tea plant transcriptome

**Fig. 4:**
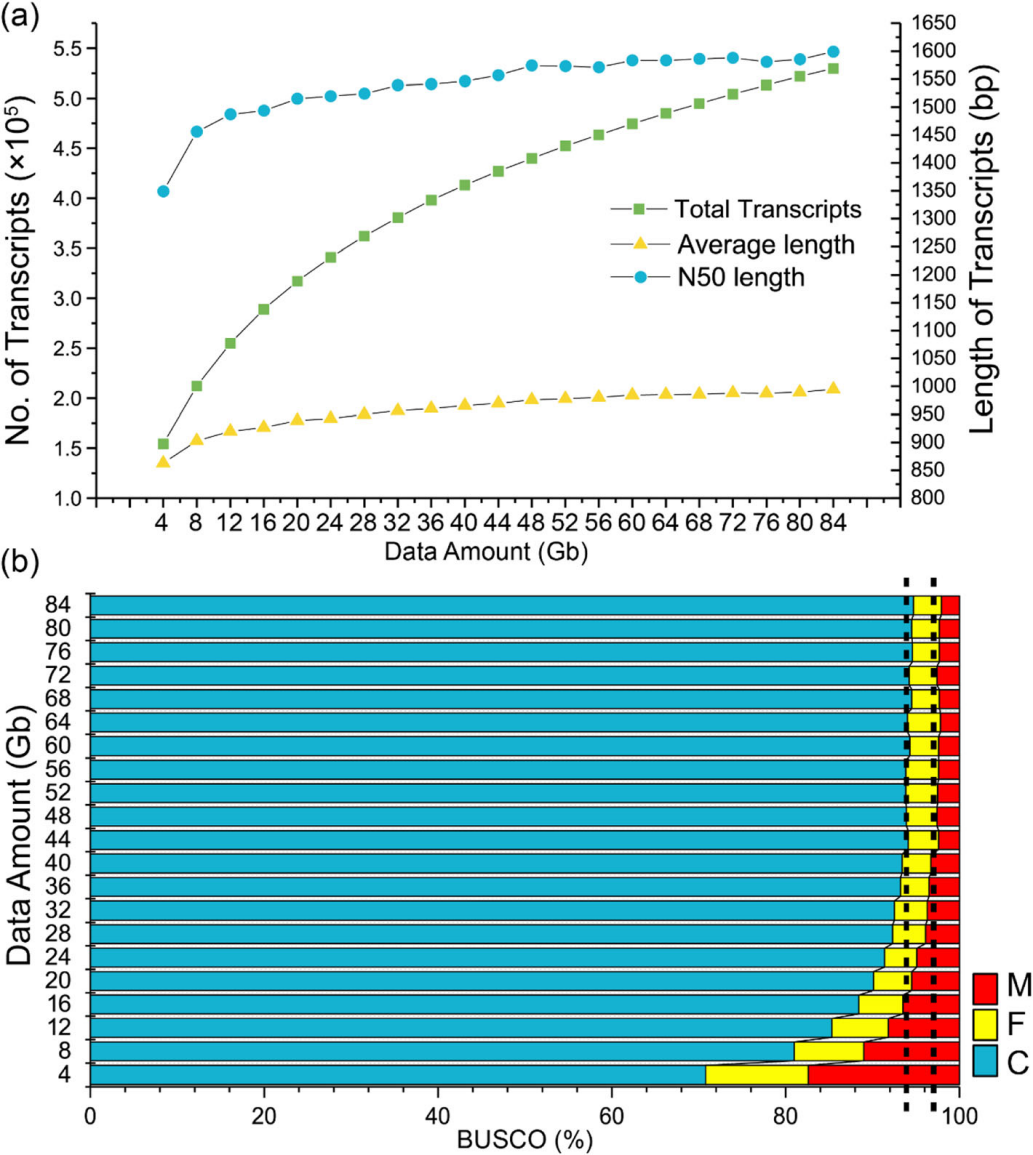
Statistic of the transcriptome assemblies using Bridger with different amount of sequencing data from replicate 1. **a** Total number and sequence length of the transcripts. The x-axis represents the total amount of the sequencing data ranging from 4 to 84 Gb; **b** BUSCO evaluation of the completeness of transcriptome assemblies. The x-axis indicates the percentage of each type of BUSCO, while the y-axis displays the transcriptome assembled using different amount of sequencing data. M: Missing BUSCOs; F: Fragmented BUSCOs; C: Complete BUSCOs

Tea plants harbor a large and structurally complex genome [15, 16]. It has not been determined how much sequencing data are actually needed to fully capture the nature of its transcription. To investigate this possibility, we simulated a total of 21 datasets from eight representative tissues of tea plants with a total data amount ranging from 4 to 84 Gb. Each dataset was a mixture of paired reads randomly extracted from sequencing data of eight tissues. We applied Bridger to assemble the datasets from each data group. The results show that, although the total number of transcripts increased with higher amounts of data, the N50 size and average sequence length of transcripts trended towards stability at 48 Gb (Fig. 4a). This finding indicates that sequencing data of 48 Gb may have reached the saturation point for tea plant transcriptome assembly. BUSCO evaluation of the completeness of transcriptome assembly further confirms the assumption. We show that the missing rate of the assembled transcriptome largely decreased from 4 to 20 Gb, then slowly declined, and finally stabilized at 48 Gb with a missing rate of 2.6% (Fig. 4b). We re-simulated 21 datasets using the same method described above and separately assembled them into transcripts using Bridger, showing a comparable assembly performance and sequence patterns with those reported above. This finding indicates a high repeatability of the obtained results (Additional file 1: Figures S5 and S6).

Tea plants are leaf-use crops of economic importance. In some cases, we merely focus on the transcriptomic dynamics of a few specific tissues or developmental stages. We simulated 88 datasets from eight tissues, 22 datasets from two tissues, and 11 datasets from three tissues (Additional file 1: Table S5~S9). The results indicate that the amount of input reads increases, the number of transcripts obtained by the assembly also increased, and the same phenomenon occurred both in single tissue de novo and multiple polled tissues. At the same input reads, assembling different tissue types will yield different numbers of transcripts. For example, at 1 Gb reads input, de novo bud, root, stem and fruit can provide almost 80,000 transcripts, but the mature leaf in summer yields less than 70,000 transcripts. Additionally, the BUSCO evaluation of 1 Gb de novo flower and root both lost over 50% of BUSCOs. When the input reads were doubled, the missing BUSCO percentage decreased slightly, and the missing rate of the four tissues was lower than 30%. When the input reads reached 6 Gb, the percentage of missing BUSCOs of four tissues was lower than 15%, and that for six of eight tissues was lower than 20%. If we continued to place more reads into the de novo assembly, the percentage of missing BUSCOs became lower in all eight tissues, and was less than 20% with 9 Gb reads input while the percentage of missing BUSCO about the first young leaf was less than 10%. When we mixed reads from two tissues or three tissues, we obtained better results compared to the same depth in single tissue de novo assembly (Additional file 1: Table S8 and S9).

### Time usage by different assemblers

The run times required for each assembler to assemble the same amount of input reads vary significantly. A suitable assembly software not only performs well in assembly quality, but also should assemble in the least time possible. We simulated three data sets (0.5 Gb, 1.0 Gb, 3 Gb) to compare the run times of five assemblers with different *k*-mer values (19, 21, 23, 25, 27, 29, 32). The *k*-mer value of Trinity is fixed to 25. We analyzed the results of run time requirements, and found large differences among the tested assembly software (Additional file 1: Table S10). Taking *k* = 25 as an example, SOAP*denovo* had the shortest assembly time, followed by Trans-ABySS. The other three assembly software required considerably more time than Trans-ABySS, with Trinity requiring the most. With an increased number of reads, the assembly time of each software also increased, though it was not by multiples as reads increased. Another phenomenon is that when the *k* was set to 19, assembly times were considerably longer than with other *k*-mer values.

## Discussion

De novo assembly of transcriptomes from short-read RNA-Seq data presents several challenges to bioinformaticians. This study was designed to evaluate the performances of five publicly available assemblers that are commonly used to assemble short-read transcriptome data: SOAP*denovo*, Trans-ABySS, Bridger, BinPacker and Trinity. To reveal the key factors to consider when choosing an optimal strategy and software tool, we set up variable testing conditions by using five assemblers using the same input reads and with single *k*-mer (25) and multiple *k*-mer (19~32). The assemblers were compared under these conditions while mapping to such factors as genome, CDS, and low coverage depth vs. high coverage depth. We measured results in terms of run time usage, transcript accuracy, integrity and completeness, and sensitivity to assembling transcripts from low to high expression levels. By analyzing and comparing the assembled results under various conditions, we were able to develop useful guidelines that may facilitate future transcriptome studies.

We found that the performances of Bridger and BinPacker were better than those of the other three assemblers, at least in the tea plant transcriptome assembly. Currently, advances in third-generation sequencing technology will accelerate the transcriptome study of several crop species [56, 57]. Accordingly, the completeness of the transcriptome generated from third-generation sequencing (e.g., PacBio SMRT) can be used as a key factor to evaluate the assembly performance of the transcriptome from next-generation sequencing. Two transcript datasets of tea plants were recently generated using PacBio SMRT sequencing technology [3, 58]. They contain 80,217 and 93,833 transcripts, respectively. Analysis of their completeness using plant BUSCOs found that the completeness of these two transcripts datasets of tea plant generated using third-generation sequencing varied significantly, ranging from 55.1 to 92.7% (Additional file 1: Table S11), with the best performance showing a similar completeness of our NGS transcriptome assembly (92.5%) with 32 Gb data. This result indicates that transcriptome assembled from sufficient NGS sequencing data (e.g., 32 Gb or more) can achieve a comparable completeness of transcriptome from third-generation sequencing technology.

In addition, although polyploidy is widespread in angiosperms, most of the current transcriptome assemblers are typically developed, refined, and tested for diploid organisms [59–64]. De novo assembly of the transcriptome of diploid plants is notably easy using the currently available assemblers. However, this approach has several unique challenges in the transcriptome assembly of polyploidy plants particularly with the large genome size, which possess a large number of paralogues, orthologues, homologues and isoforms that are difficult to disentangle at the sequence level [65–67]. All of the assembler assessments and parameter tests in the present study are optimal for diploid plants (e.g., tea plants). However in the case of better constructing the transcriptome of polyploidy plants, we believe that future research is warranted to 1) prepare high-quality material and extract high-quality non-fragmented RNA; 2) obtain adequate depth of sequencing reads with high-quality and long sequence length (e.g., PacBio SMRT); and (3) develop suitable software/pipeline that can efficiently discriminate homologue nucleotide differences and errors among the sequencing data. This research may eventually help to resolve the transcriptome complexity of polyploidy plants [68, 69].

It is widely accepted that most plants (e.g., tea plants) have undergone multiple whole genome duplication events that accordingly duplicate their protein-coding genes [70, 71]. This largely challenged the duplicated genes (transcripts) construction with the current transcriptome assemblers, as almost all of the assemblers are difficult to distinguish the origin of duplicated transcripts at the sequence level (e.g., PCR amplification, assembly redundancy, alternative splicing or even assembly errors) [72, 73]. In the present study, we used the BUSCO pipeline to evaluate the proportion of duplicated transcripts recovered by different assemblers. The results show that approximately 40% of duplicated genes were moderately constructed by the five assemblers. Compared to the other three assemblers, Bridger and BinPacker showed high performance in duplicate gene reconstruction. Transcripts with short or long sizes also presents considerably huge difficulties to the current de novo transcriptome assemblers. The short transcripts are always truncated and weakly expressed, as they usually lack sufficient sequencing reads and evidence for transcript reconstruction. As reported previously [17, 44], the annotation efficiency of the NR database was negatively related to the length of assembled transcripts. For example, over 80% of transcripts are not able to find any homologues in the NR database if their sequence length is < 500 bp. Similar situations also occurred in the transcripts with long sequence lengths. The long transcripts may be derived from the over-assembly of nearby transcripts. In particular, this situation is highly common in the transcriptome assembly of fungal species, as most of the fungal species always have high gene density [74, 75]. For plant species, the average proportion of long transcripts (> 5 kb) from transcriptome assembly is approximately 2% [14]. Most of these transcripts (~ 60%) are confirmed to be aligned with multiple homologues of the NR database, showing incorrect linking during the assembly. Therefore, most of the current de novo assemblers may possess poor abilities to resolve the duplicated transcripts, as well as some notably short or long transcripts, which should receive more attention in transcriptome studies in the future.

## Conclusions

In this study, we comprehensively evaluated the de novo transcriptomic assemblies of tea plants with different amounts of RNA-seq data employing five well-known assemblers. Although the total number of assembled transcripts increased with the growing amount of sequencing data, the proportion of unassembled transcripts became saturated as revealed by plant BUSCO datasets. The assembler Bridger and BinPacker performed better in the tea plant transcriptome assembly, which also harbored the shorter running time. Nevertheless, the Bridger package shows the best performance in both assembly completeness and accuracy, as evaluated using the BUSCO datasets and genomic alignments. This study will be of significance in helping the tea research community gain better sequencing and assembly of tea plant transcriptomes under conditions of interest, and thus help to answer the major biological questions currently facing the tea industry.

## Materials and methods

### Plant material

The 6-year-old tea plant (*C. sinensis* var. *sinensis* cv. shuchazao) used in this study were planted in the DeChang Tea Plantation of Anhui, China. Eight tissues, including apical bud, first young leaf, second young leaf, mature leaf in summer, stem, flower, fruit, and root were sampled and stored at − 80 °C.

### RNA isolation, transcriptomic library construction and RNA sequencing

As described in a previous study [51], total RNA was extracted separately from the 8 tissues using a modified CTAB method in triplicate. The yield and quality of RNA were determined by agarose gel electrophoresis and by measuring with a Nanodrop 2000. Equal amounts of RNA from three different samples were pooled before cDNA library preparation. The mRNA enrichment, cDNA synthesis, fragmentation, adapter addition, selection of fragment size, PCR amplification, and transcriptomic sequencing were performed according to the manufacturer’s protocol (Illumina, CA, USA). The cDNA library was examined using an Agilent 2100 Bioanalyzer prior to sequencing on an Illumina HiSeq 2000 platform with a read length of 90 bp.

### Data preprocessing and de novo assembly

Preliminary screening was performed on raw sequencing reads to remove low-quality reads and reads with adaptor sequences using the Trimmomatic (version 0.32) program [76]. Only reads with a trimmed length over 30 bp were used in further analyses. The number of paired-end reads in each sample is shown in Additional file 1: Table S2. The remaining reads were then adopted for de novo assembly using the following assemblers with default parameters: Trinity (release-20,130,225) [6], Bridger (2014-12-01) [7], SOAP*denovo* (ver. 1.04) [8], Trans-ABySS (ver. 1.55) [9], and BinPacker (ver. 1.0) [10].

The simulated data of different sequencing depths were subsampled from the real data derived from the 8 samples using a Perl script. We tested four de novo models: the first is to assemble 32 Gb reads by randomly choosing 4 Gb per tissue; the second use simulation method to generate 4 Gb (0.5 Gb per tissue) to 84 Gb (10.5 Gb per tissue) reads. The third model is to assemble transcripts from 1 to 11 Gb (1 Gb per step) reads per sample. The fourth model is to assemble two or three samples from different tissue combinations, such as one bud and leaf, one leaf and root, one bud and two leaves.

### Mapping reconstructed transcripts to genomic and coding sequences

Genome sequence and gene models for tea plants were downloaded from the Tea Plant Information Archive (http://tpia.teaplant.org), and used to evaluate the performance of each assembler [77]. BLAT [78] with default parameters was applied to map the reconstructed transcripts from each assembler to non-identical reference coding sequences and reference genomes. Four groups of hits were classified to evaluate the capability for CDS reconstruction: 1) Coverage of the entire reference coding sequence without mismatches, insertions or deletions (98%); 2, 3, 4) At least 95%/80%/50% of sequence alignment identity covering the entire reference coding sequence. To assess the accuracy of reconstructed transcripts, we also aligned them to the reference genome using BLAT, and accuracy was assessed using the numbers of reconstructed transcripts that had at least 50% of their lengths aligned back to genome. Transcripts with less than 50% of their lengths mapped back to the genome were defined as unmapped transcripts. The Tbtools [79] software was used to plot the graphical heat map.

### Evaluation of assembly quality using BUSCO

For further quantitative assessment of the assembly completeness, we applied BUSCO [80] with default settings. The BUSCO tool analyzed each transcriptome assembly state with complete and single-copy BUSCOs, complete and duplicated BUSCOs, fragmented BUSCOs, and missing BUSCOs using the embryophyta_odb9 database, which contains 1440 total BUSCO groups.

### Calculation of gene expression

The expression levels of transcripts were calculated using the FPKM method. First, reads were mapped to transcript datasets using Bowtie2 (version 2.1.0) [81] in a sensitive setting, and then FPKM values for each transcripts were subsequently calculated by RESM (version 1.2.29) [82].

## Supplementary information

**Additional file 1: Figure S1.** Venn diagram shows the transcripts intersected in different assemblers. **Figure S2.** Transcripts mapped to coding sequences and genome sequences with different coverage and identity thresholds. **Figure S3.** Assembly quality of transcripts at different expression levels. **Figure S4.** Completeness of the assembled transcripts at different expression levels. **Figure S5.** Comparison of the assembly performances between two replications of datasets randomly selected from eight representative tissues of tea plant. **Figure S6.** Statistic of the transcriptome assemblies using Bridger with different amount of sequencing data from replicate 2. **Table S1.** Summary of transcriptome assemblies of tea plant in previous studies. **Table S2.** Summary of the data used in this study. **Table S3.** Coverage of transcripts mapped to the reference genome. **Table S4.** Statistic of the Bridger assembly using different *k*-mer values. (a) Assembly characteristics; (b) completeness assessment using BUSCO; (c) length distribution. **Table S5.** Statistics of assembly. (a) Apical bud; (b) flower; (c) fruit; (d) second young leaf; (e) mature leaf in summer; (f) first young leaf; (g) root; (h) stem. **Table S6.** Length distribution of assembly. (a) Apical bud; (b) flower; (c) fruit, (d) second young leaf; (e) mature leaf in summer; (f) first young leaf; (g) root; (h) stem. **Table S7.** BUSCO evaluation of assembly. (a) Apical bud; (b) flower; (c) fruit; (d) second young leaf; (e) mature leaf in summer; (f) first young leaf; (g) root; (h) stem. **Table S8.** Statistic of assembly. (a) Apical bud and first young leaf; (b) apical bud and root. **Table S9.** Statistic of pooled assembly. (a) Assembly characteristics; (b) length distribution; (c) BUSCO evaluation. **Table S10.** Runtime (hours) performance for each assembler with different amount of input data. (a) 0.5 Gb; (b) 1 Gb; (c) 3 Gb. **Table S11.** Completeness of transcriptomes generated in tea plant using PacBio technology.

## Data Availability

The Illumina RNA-seq data is available in the NCBI SRA database under the accessions of SRR1928149 and SRP056466.

## Abbreviations

BUSCO: Benchmarking universal single-copy orthologs
CDS: Coding sequence
FL: Full length
RNA-seq: RNA sequencing
