# Supplementary material for: Optimized sequencing depth and de novo assembler for deeply reconstructing the transcriptome of the tea plant, an economically important plant species
Source: BMC Bioinformatics. 2019 Nov 6;20:553. doi: 10.1186/s12859-019-3166-x (PMC6836513; doi:10.1186/s12859-019-3166-x)
Supplement: Supplementary file 1 — Additional file 1: Figure S1. Venn diagram shows the transcripts intersected in different assemblers. Figure S2. Transcripts mapped to coding sequences and genome sequences with different coverage and identity thresholds. Figure S3. Assembly quality of transcripts at different expression levels. Figure S4. Completeness of the assembled transcripts at different expression levels. Figure S5. Comparison of the assembly performances between two replications of datasets randomly selected from eight representative tissues of tea plant. Figure S6. Statistic of the transcriptome assemblies using Bridger with different amount of sequencing data from replicate 2. Table S1. Summary of transcriptome assemblies of tea plant in previous studies. Table S2. Summary of the data used in this study. Table S3. Coverage of transcripts mapped to the reference genome. Table S4. Statistic of the Bridger assembly using different k-mer values. (a) Assembly characteristics; (b) completeness assessment using BUSCO; (c) length distribution. Table S5. Statistics of assembly. (a) Apical bud; (b) flower; (c) fruit; (d) second young leaf; (e) mature leaf in summer; (f) first young leaf; (g) root; (h) stem. Table S6. Length distribution of assembly. (a) Apical bud; (b) flower; (c) fruit, (d) second young leaf; (e) mature leaf in summer; (f) first young leaf; (g) root; (h) stem. Table S7. BUSCO evaluation of assembly. (a) Apical bud; (b) flower; (c) fruit; (d) second young leaf; (e) mature leaf in summer; (f) first young leaf; (g) root; (h) stem. Table S8. Statistic of assembly. (a) Apical bud and first young leaf; (b) apical bud and root. Table S9. Statistic of pooled assembly. (a) Assembly characteristics; (b) length distribution; (c) BUSCO evaluation. Table S10. Runtime (hours) performance for each assembler with different amount of input data. (a) 0.5 Gb; (b) 1 Gb; (c) 3 Gb. Table S11. Completeness of transcriptomes generated in tea plant using PacBio technology. [file 12859_2019_3166_MOESM1_ESM.zip › Supplementary Materials2.docx]

**Supplementary Materials**

Figure S1. Venn diagram shows the transcripts intersected in different assemblers.

Figure S2. Transcripts mapped to Coding sequences and Genome sequences with different coverage and identity thresholds.

Figure S3. Assembly quality of transcripts at different expression levels.

Figure S4. Completeness of the assembled transcripts at different expression levels.

Figure S5. Comparison of the assembly performances between two replications of datasets randomly selected from eight representative tissues of tea plant.

Figure S6. Statistic of the transcriptome assemblies using Bridger with different amount of sequencing data from replicate 2.

Table S1. Summary of transcriptome assemblies of tea plant in previous studies.

Table S2. Summary of the data used in this study.

Table S3. Coverage of transcripts mapped to the reference genome.

Table S4. Statistic of the Bridger assembly using different *k*-mer values. (a) Assembly characteristics; (b) completeness assessment using BUSCO; (c) length distribution.

Table S5. Statistics of assembly. (a) Apical bud; (b) flower; (c) fruit; (d) second young leaf; (e) mature leaf in summer; (f) first young leaf; (g) root; (h) stem.

Table S6. Length distribution of assembly. (a) Apical bud; (b) flower; (c) fruit; (d) second young leaf; (e) mature leaf in summer; (f) first young leaf; (g) root; (h) stem.

Table S7. BUSCO evaluation of assembly. (a) Apical bud; (b) flower; (c) fruit; (d) second young leaf; (e) mature leaf in summer; (f) first young leaf; (g) root; (h) stem.

Table S8. Statistic of assembly. (a) Apical bud and first young leaf; (b) apical bud and root.

Table S9. Statistic of pooled *de novo* assembled from apical bud, first young leaf and second young leaf. (a) Assembly characteristics; (b) length distribution; (c) BUSCO evaluation.

Table S10. Runtime (hours) performance for each assembler with different amount of input data. (a) 0.5 Gb; (b) 1 Gb; (c) 3 Gb.

Table S11. Completeness of transcriptomes generated in tea plant using PacBio technology.

**
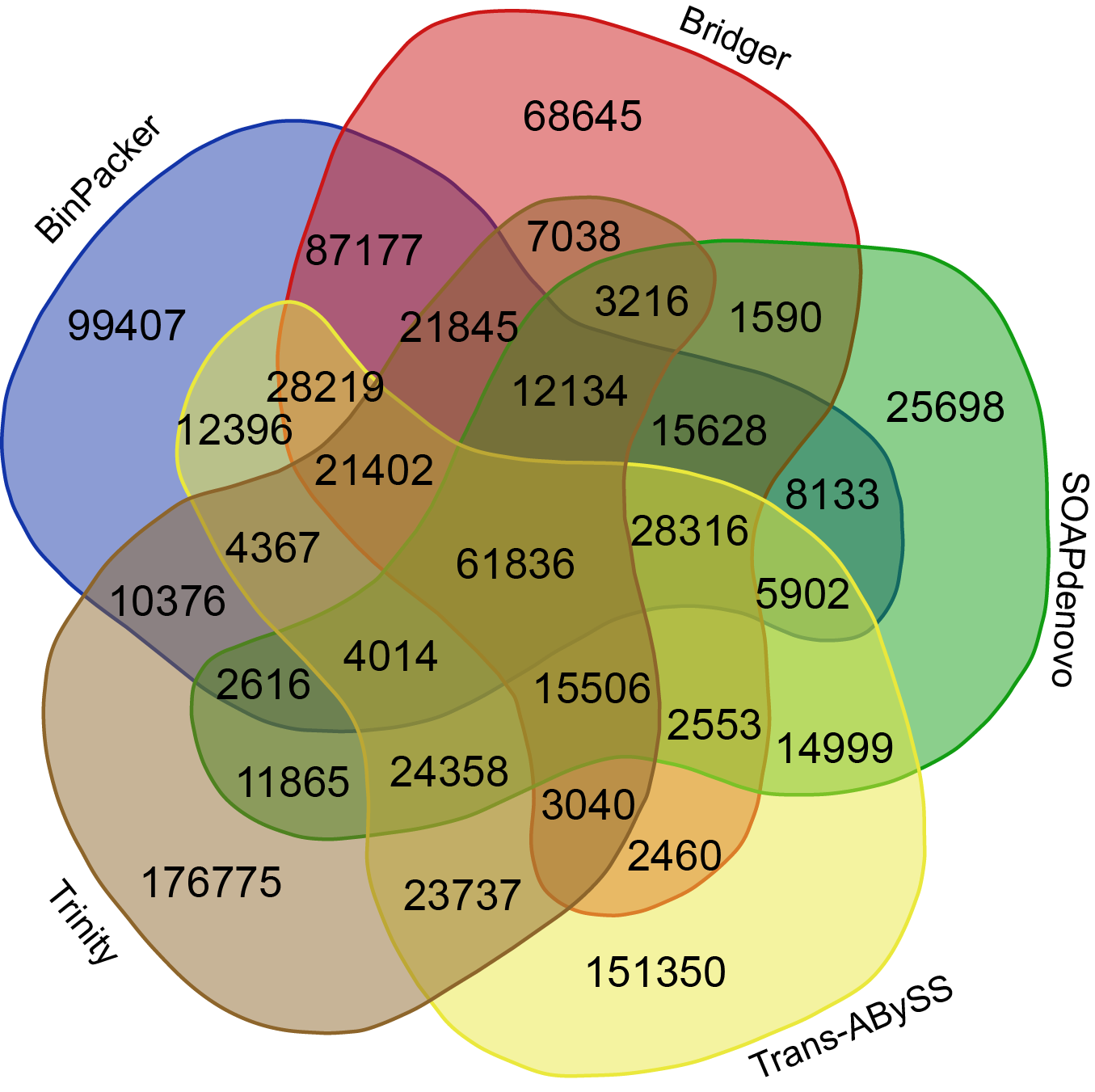
**

**Figure S1. Venn diagram shows the transcripts intersected in different assemblers.**

**
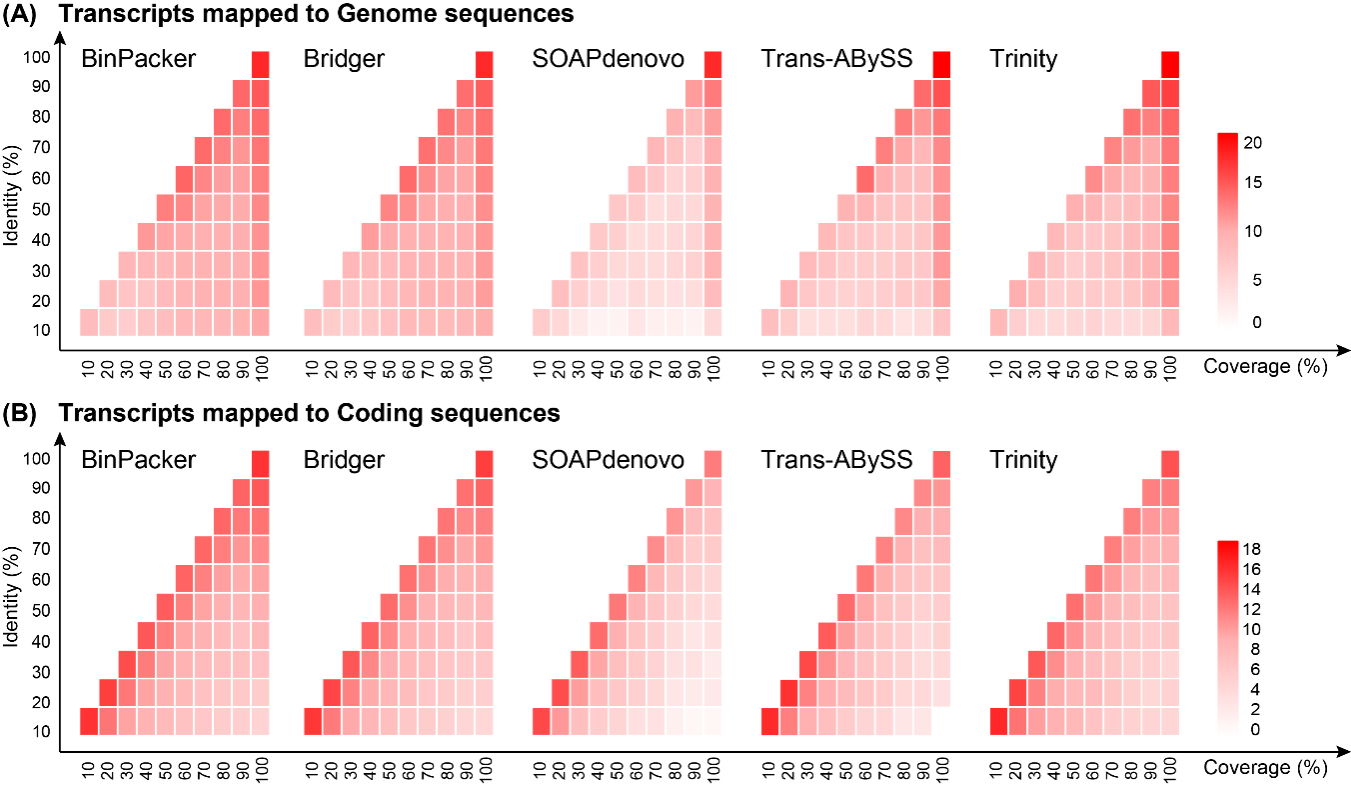
**

**Figure S2. Transcripts mapped to coding sequences and genome sequences with different coverage and identity thresholds.**

**
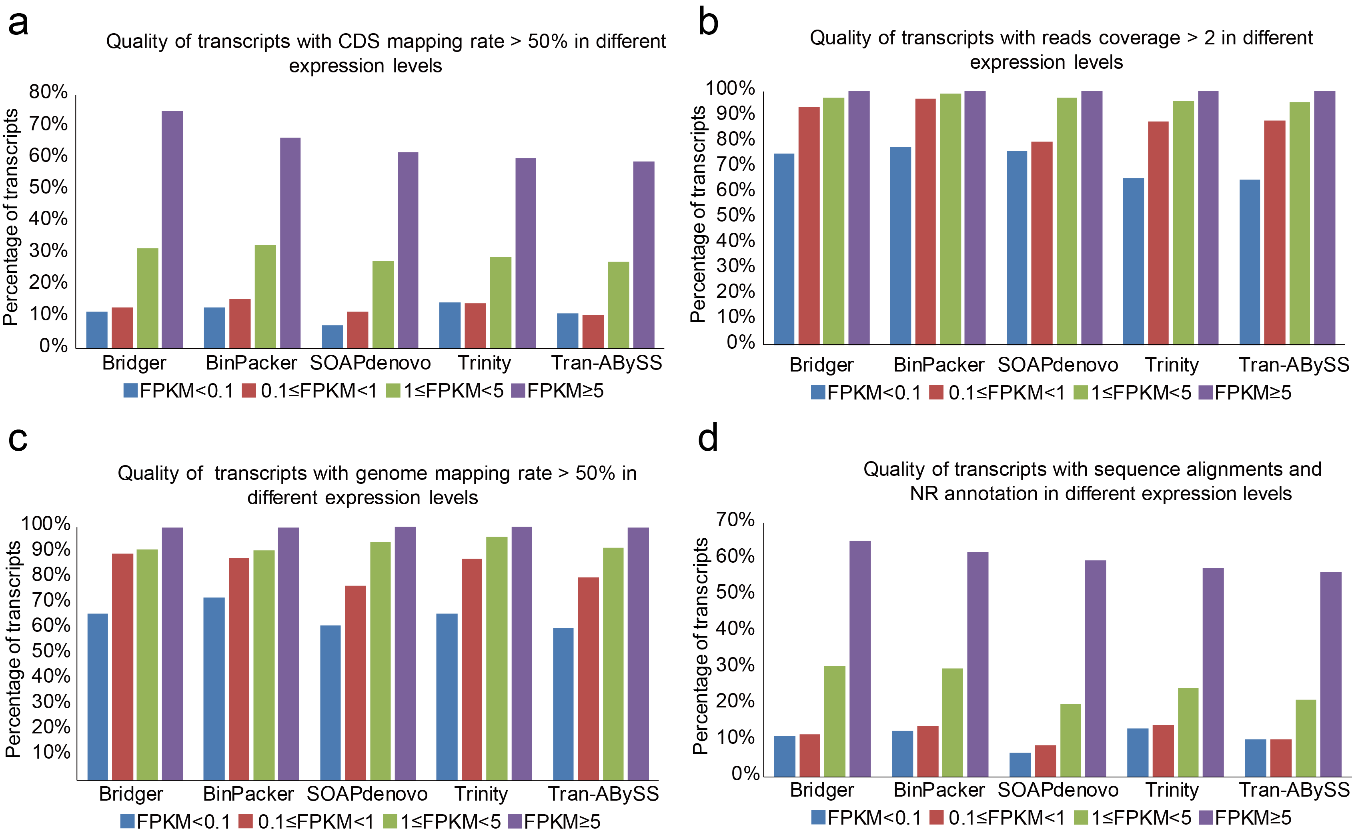
**

**Figure S3. Assembly quality of transcripts at different expression levels.** (d) Quality of transcripts with reads coverage > 2, genome mapping rate > 50%, CDS mapping rate > 60% and NR annotation in different expression levels.

**
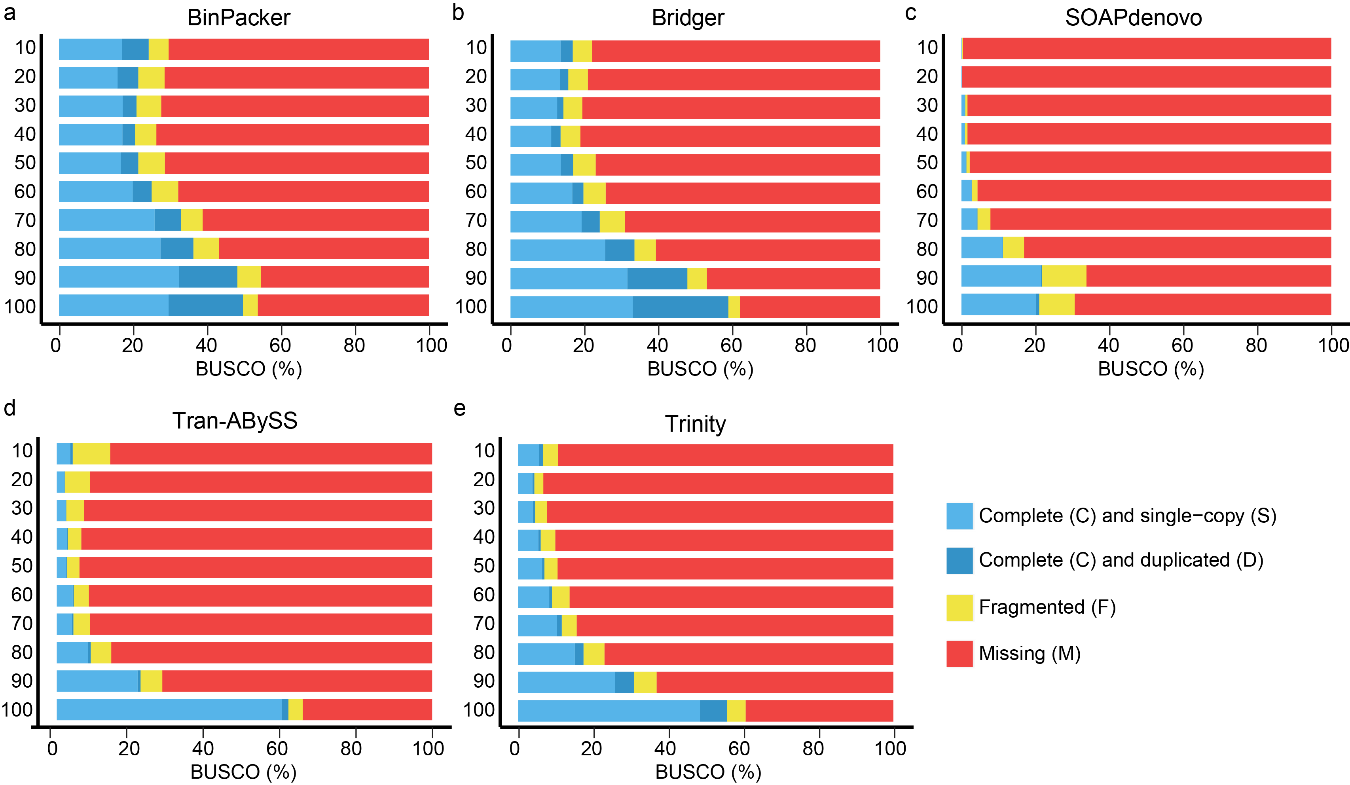
**

**Figure S4. Completeness of the assembled transcripts at different expression levels.** The x-axis indicates percentage of BUSCO evaluation, while the y-axis represents the transcripts at different expression quintiles.

**
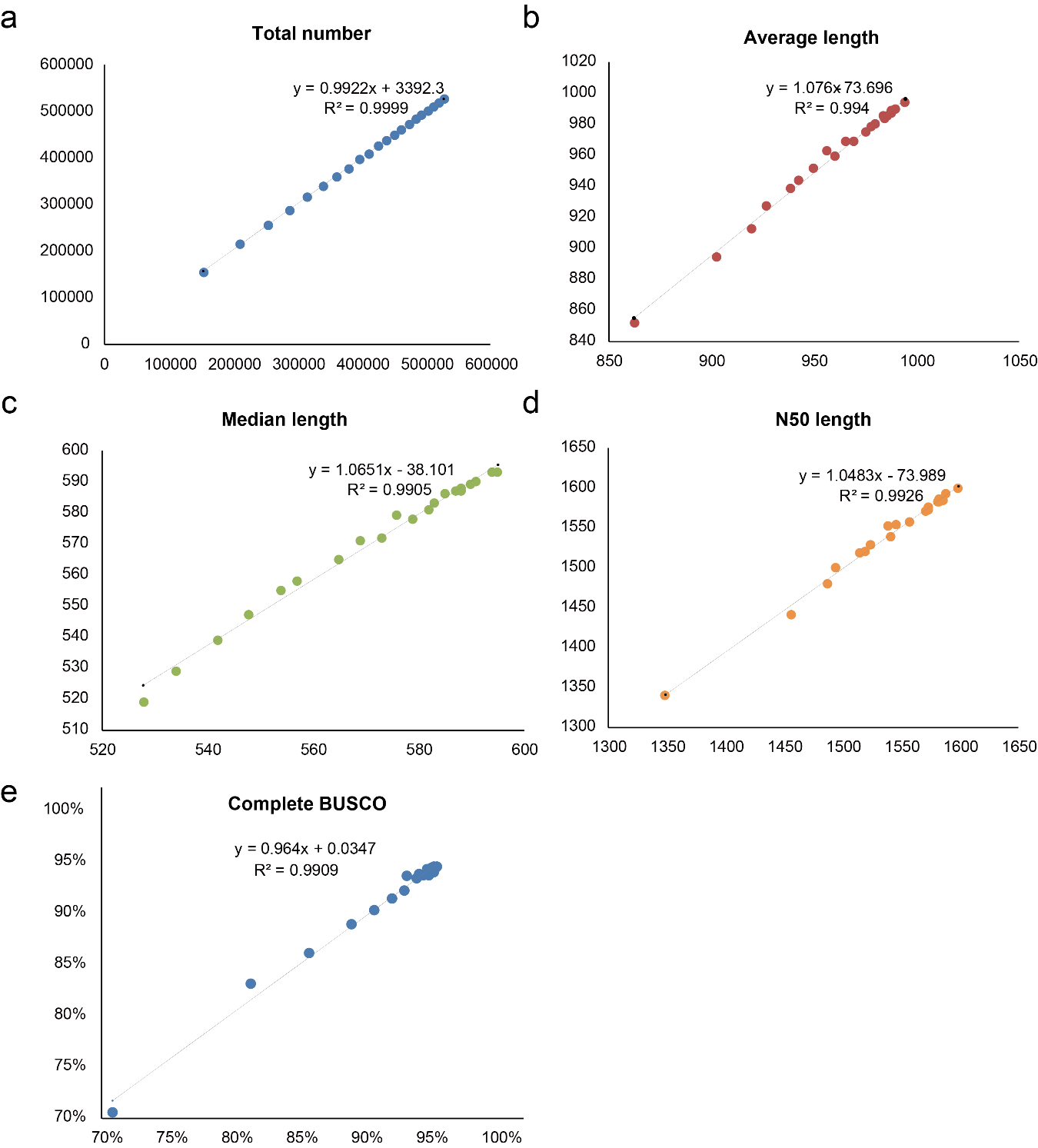
**

**Figure S5. Comparison of the assembly performances between two replications of datasets randomly selected from eight representative tissues of tea plant.** X-axis and y-axis represent the transcriptomes assembled from 21 datasets, ranging from 4 – 84 Gb, of replicate 1 and 2, respectively. The assembly performance between these two replicates are compared on (a) total number; (b) average length; (c) median length; (d) N50 length; and (e) complete BUSCO. Correlation analysis of the two replicates were calculated by linear correlation analysis.

**
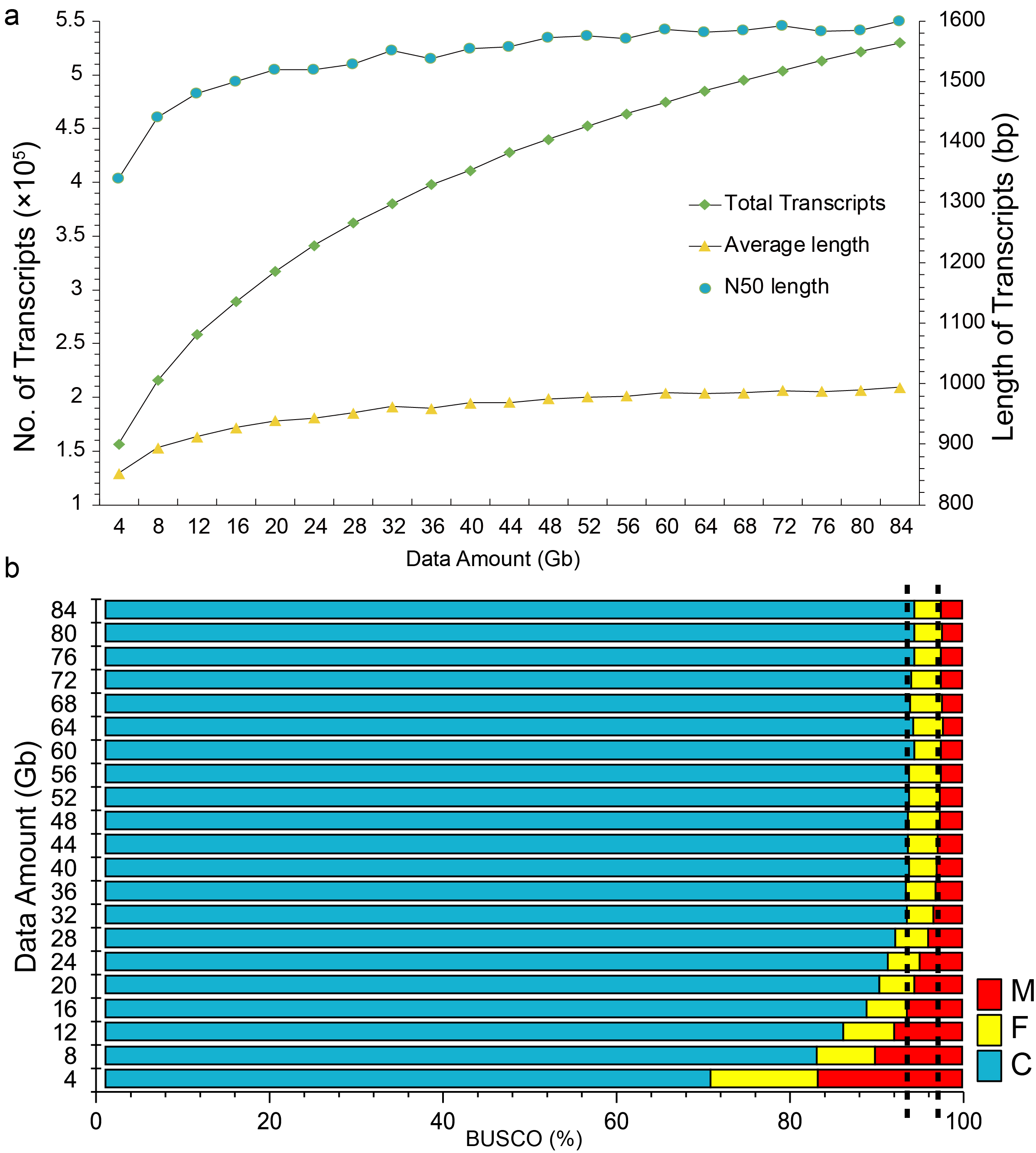
**

**Figure S6. Statistic of the transcriptome assembly using Bridger with different amount of sequencing data from replicate 2.** (a) Overview of the transcripts assembled from replicate 2 datasets, the x-axis represents the transcriptome assembled from different amount of the sequencing data ranging from 4 Gb to 84 Gb; (b) BUSCO evaluation of the completeness of transcriptome assembly from replicate 2 datasets, the x-axis indicates the percentage of each type of BUSCO, while the y-axis displays the transcriptome assembled using different amount of sequencing data.

| **Table S2. Summary of the data used in this study.** | | | | | | |
| --- | --- | --- | --- | --- | --- | --- |
| **Sample** | **Raw Reads** | **Clean Reads** | **Clean bases (bp)** | **Q20 %** | **N %** | **GC %** |
| **CS-AB** | 150,084,806 | 135,688,092 | 12,211,928,280 | 97.44% | 0.01% | 46.06% |
| **CS-FL** | 149,419,990 | 138,110,716 | 12,429,964,440 | 97.57% | 0.01% | 46.61% |
| **CS-FR** | 139,099,682 | 127,972,068 | 11,517,486,120 | 97.55% | 0.00% | 46.20% |
| **CS-FYL** | 138,242,956 | 124,627,402 | 11,216,466,180 | 97.04% | 0.01% | 46.01% |
| **CS-SYL** | 131,264,800 | 119,864,740 | 10,787,826,600 | 97.22% | 0.01% | 45.59% |
| **CS-ML** | 146,626,208 | 133,506,560 | 12,015,590,400 | 97.57% | 0.00% | 45.94% |
| **CS-R** | 149,788,850 | 137,194,326 | 12,347,489,340 | 97.20% | 0.01% | 46.46% |
| **CS-S** | 141,143,966 | 128,659,082 | 11,579,317,380 | 97.22% | 0.01% | 45.95% |
| **Total** |  | 1,045,622,986 | 94,106,068,740 | - | - | - |

| **Table S3. Coverage of transcripts mapped to the reference genome.** | | | | | |
| --- | --- | --- | --- | --- | --- |
|  | **All Transcripts** | **coverage<50%*** | **coverage<50*** | **coverage>50*** | **coverage>50*** |
| **Bridger** | 360789 | 11347 | 0.031450515 | 349442 | 0.968549 |
| **BinPacker** | 413206 | 14247 | 0.03447917 | 398959 | 0.965521 |
| **Trans-ABySS** | 383149 | 1825 | 0.00476316 | 381324 | 0.995237 |
| **SOAP***denovo* | 221539 | 1015 | 0.004581586 | 220524 | 0.995418 |
| **Trinity** | 373019 | 2338 | 0.006267777 | 370681 | 0.993732 |
| * Coverage (%) indicates the percentage of the mapped transcripts in all the assembled transcripts. | | | | | |

| **Table S4a. Assembly statistics at different *k*-mer values in Bridger assembly.** | | | | | | |  |
| --- | --- | --- | --- | --- | --- | --- | --- |
|  | **Total transcripts** | **Total bases** | **Maximum length** | **Average length** | **N50 length** |  | |
| **Bridger-k19** | 273893 | 274854010 | 27833 | 1003.51 | 1531 |  | |
| **Bridger-k21** | 350330 | 346120743 | 28766 | 987.98 | 1554 |  | |
| **Bridger-k23** | 370677 | 351703799 | 18579 | 948.81 | 1524 |  | |
| **Bridger-k25** | 380605 | 364039338 | 22041 | 956.48 | 1539 |  | |
| **Bridger-k27** | 383138 | 364547394 | 20166 | 951.48 | 1524 |  | |
| **Bridger-k29** | 383689 | 364629474 | 19347 | 950.33 | 1513 |  | |
| **Bridger-k32** | 390761 | 350354909 | 19996 | 896.6 | 1430 |  | |
| **Table S4b. BUSCO at different *k*-mer values in Bridger assembly.** | | | | | |  | |
|  | **C** | **S** | **D** | **F** | **M** |  | |
| **Bridger-k19** | 56.10% | 23.50% | 32.60% | 22.30% | 21.60% |  | |
| **Bridger-k21** | 86.70% | 18.40% | 68.30% | 6.70% | 6.60% |  | |
| **Bridger-k23** | 91.20% | 15.70% | 75.50% | 4.40% | 4.40% |  | |
| **Bridger-k25** | 92.50% | 14.90% | 77.60% | 3.80% | 3.70% |  | |
| **Bridger-k27** | 93.00% | 15.30% | 77.70% | 3.30% | 3.70% |  | |
| **Bridger-k29** | 86.70% | 18.40% | 68.30% | 6.70% | 6.60% |  | |
| **Bridger-k32** | 92.70% | 15.20% | 77.50% | 3.30% | 4.00% |  | |
| **Table S4c. Length distribution at different *k*-mer values in Bridger assembly.** | | | | | | |  |
|  | **200-500** | **500-1000** | **1000-1500** | **1500-2000** | **2000-3000** | **>3000** | |
| **Bridger-k19** | 110721 | 79829 | 35571 | 17596 | 15776 | 14400 | |
| **Bridger-k21** | 147160 | 96748 | 43124 | 23038 | 21452 | 18808 | |
| **Bridger-k23** | 166243 | 99054 | 41496 | 22449 | 22606 | 18829 | |
| **Bridger-k25** | 169453 | 100762 | 43286 | 24181 | 23513 | 19410 | |
| **Bridger-k27** | 170813 | 100847 | 43979 | 24389 | 24331 | 18779 | |
| **Bridger-k29** | 170192 | 101278 | 44492 | 24800 | 24623 | 18304 | |
| **Bridger-k32** | 185077 | 100809 | 41875 | 23579 | 22831 | 16590 | |

| **Table S5a. Statistics of each *de novo* assembly (apical bud)** | | | | | | |
| --- | --- | --- | --- | --- | --- | --- |
|  | **Total Sequences** | **Total bases** | **Max length** | **Average length** | **Median length** | **N50** |
| **1Gb** | 80574 | 54728471 | 11850 | 679.23 | 465 | 916 |
| **2Gb** | 107826 | 84380124 | 15623 | 782.56 | 509 | 1144 |
| **3Gb** | 128301 | 106627076 | 12254 | 831.07 | 523 | 1271 |
| **4Gb** | 144782 | 123794391 | 14160 | 855.04 | 529 | 1328 |
| **5Gb** | 159770 | 138513153 | 15423 | 866.95 | 526 | 1365 |
| **6Gb** | 173494 | 152447036 | 15992 | 878.69 | 526 | 1405 |
| **7Gb** | 185931 | 164543248 | 20233 | 884.97 | 526 | 1421 |
| **8Gb** | 197429 | 176215607 | 26759 | 892.55 | 527 | 1444 |
| **9Gb** | 208102 | 186679426 | 22211 | 897.06 | 526 | 1453 |
| **10Gb** | 217810 | 196039975 | 20879 | 900.05 | 530 | 1461 |
| **11Gb** | 226677 | 205063230 | 22212 | 904.65 | 531 | 1471 |
| **Table S5b. Statistic of each *de novo* assembly (flower)** | | | |  |  |  |
|  | **Total Sequences** | **Total bases** | **Max length** | **Average length** | **Median length** | **N50** |
| **1Gb** | 73293 | 48421595 | 8042 | 660.66 | 431 | 914 |
| **2Gb** | 99117 | 73790102 | 10274 | 744.47 | 460 | 1122 |
| **3Gb** | 119298 | 93010126 | 12967 | 779.65 | 466 | 1211 |
| **4Gb** | 136315 | 109079788 | 14928 | 800.2 | 472 | 1261 |
| **5Gb** | 151009 | 123146062 | 15853 | 815.49 | 474 | 1307 |
| **6Gb** | 163936 | 135066274 | 14936 | 823.9 | 476 | 1332 |
| **7Gb** | 176201 | 146242054 | 17409 | 829.97 | 480 | 1340 |
| **8Gb** | 187606 | 157318147 | 18933 | 838.56 | 482 | 1362 |
| **9Gb** | 197998 | 166634959 | 19244 | 841.6 | 485 | 1367 |
| **10Gb** | 207487 | 175968377 | 23227 | 848.09 | 487 | 1378 |
| **11Gb** | 216949 | 184505191 | 22236 | 850.45 | 490 | 1384 |
| **Table S5c. Statistic of each *de novo* assembly (fruit)** | | | |  |  |  |
|  | **Total Sequences** | **Total bases** | **Max length** | **Average length** | **Median length** | **N50** |
| **1Gb** | 80436 | 55411586 | 12785 | 688.89 | 457 | 947 |
| **2Gb** | 110768 | 85985092 | 19155 | 776.26 | 490 | 1149 |
| **3Gb** | 132660 | 108872556 | 15584 | 820.69 | 505 | 1251 |
| **4Gb** | 150913 | 128319500 | 14532 | 850.29 | 513 | 1324 |
| **5Gb** | 167027 | 145078115 | 20114 | 868.59 | 518 | 1367 |
| **6Gb** | 181140 | 159111982 | 18945 | 878.39 | 522 | 1393 |
| **7Gb** | 193382 | 171734857 | 19357 | 888.06 | 523 | 1422 |
| **8Gb** | 205339 | 184233282 | 24597 | 897.22 | 526 | 1444 |
| **9Gb** | 215770 | 195235381 | 25568 | 904.83 | 531 | 1456 |
| **10Gb** | 225613 | 204661845 | 26481 | 907.14 | 532 | 1456 |
| **11Gb** | 234978 | 214883669 | 26884 | 914.48 | 535 | 1469 |
| **Table S5d. Statistic of each *de novo* assembly (second young leaf)** | | | | | |  |
|  | **Total Sequences** | **Total bases** | **Max length** | **Average length** | **Median length** | **N50** |
| **1Gb** | 76565 | 55302110 | 12507 | 722.29 | 486 | 1005 |
| **2Gb** | 103111 | 84926634 | 13838 | 823.64 | 528 | 1233 |
| **3Gb** | 123556 | 106986275 | 16057 | 865.89 | 538 | 1340 |
| **4Gb** | 140940 | 125014137 | 17588 | 887 | 540 | 1399 |
| **5Gb** | 157017 | 141617094 | 21242 | 901.92 | 543 | 1437 |
| **6Gb** | 171054 | 155532497 | 24663 | 909.26 | 543 | 1460 |
| **7Gb** | 184023 | 167414185 | 21508 | 909.75 | 542 | 1468 |
| **8Gb** | 196205 | 178939146 | 24451 | 912 | 541 | 1466 |
| **9Gb** | 207309 | 189980152 | 24451 | 916.41 | 541 | 1482 |
| **10Gb** | 217874 | 199812023 | 24663 | 917.1 | 542 | 1483 |
| **11Gb** | 225271 | 206958305 | 24663 | 918.71 | 542 | 1486 |
| **Table S5e. Statistic of each *de novo* assembly (mature leaf in summer)** | | | | | | |
|  | **Total Sequences** | **Total bases** | **Max length** | **Average length** | **Median length** | **N50** |
| **1Gb** | 69823 | 49596963 | 12386 | 710.32 | 469 | 995 |
| **2Gb** | 99103 | 78822449 | 14879 | 795.36 | 499 | 1191 |
| **3Gb** | 121197 | 100689312 | 20109 | 830.79 | 508 | 1282 |
| **4Gb** | 140376 | 119944007 | 24594 | 854.45 | 514 | 1344 |
| **5Gb** | 156632 | 136237558 | 23785 | 869.79 | 519 | 1378 |
| **6Gb** | 171175 | 150320125 | 17556 | 878.17 | 521 | 1399 |
| **7Gb** | 184782 | 163713263 | 24847 | 885.98 | 525 | 1415 |
| **8Gb** | 196718 | 175275786 | 26067 | 891 | 527 | 1422 |
| **9Gb** | 208448 | 186818679 | 25322 | 896.24 | 529.5 | 1435 |
| **10Gb** | 218885 | 196558611 | 25317 | 898 | 532 | 1436 |
| **11Gb** | 228502 | 206166444 | 25122 | 902.25 | 533 | 1439 |
| **Table S5f. Statistic of each *de novo* assembly (first young leaf)** | | | | | | |
|  | **Total Sequences** | **Total bases** | **Max length** | **Average length** | **Median length** | **N50** |
| **1Gb** | 73659 | 52783766 | 22195 | 716.6 | 491 | 986 |
| **2Gb** | 98233 | 80684014 | 13976 | 821.35 | 541 | 1217 |
| **3Gb** | 116862 | 101607576 | 24194 | 869.47 | 553 | 1328 |
| **4Gb** | 132717 | 118346575 | 24193 | 891.72 | 558 | 1389 |
| **5Gb** | 146254 | 132504797 | 24297 | 905.99 | 560 | 1432 |
| **6Gb** | 158765 | 146380360 | 24432 | 921.99 | 563 | 1470 |
| **7Gb** | 169948 | 157859024 | 31464 | 928.87 | 562 | 1493 |
| **8Gb** | 180838 | 168271424 | 32634 | 930.51 | 559 | 1502 |
| **9Gb** | 190769 | 178716627 | 30679 | 936.82 | 561 | 1520 |
| **10Gb** | 199816 | 187352961 | 26903 | 937.63 | 560 | 1516 |
| **11Gb** | 208270 | 195541150 | 24277 | 938.88 | 561 | 1522 |
| **Table S5g. Statistic of each *de novo* assembly (root)** | | | | |  |  |
|  | **Total Sequences** | **Total bases** | **Max length** | **Average length** | **Median length** | **N50** |
| **1Gb** | 81228 | 52724090 | 24076 | 649.09 | 431 | 878 |
| **2Gb** | 110648 | 82176832 | 24273 | 742.69 | 471 | 1084 |
| **3Gb** | 130920 | 103759647 | 24540 | 792.54 | 488 | 1204 |
| **4Gb** | 148453 | 121875691 | 19145 | 820.97 | 495 | 1274 |
| **5Gb** | 163708 | 137251199 | 19145 | 838.39 | 499 | 1320 |
| **6Gb** | 177563 | 151010084 | 19495 | 850.46 | 500 | 1360 |
| **7Gb** | 190314 | 163442753 | 19496 | 858.81 | 502 | 1381 |
| **8Gb** | 202456 | 174105539 | 36238 | 859.97 | 500 | 1390 |
| **9Gb** | 213677 | 184495570 | 24541 | 863.43 | 501 | 1401 |
| **10Gb** | 224052 | 194046764 | 24708 | 866.08 | 501 | 1406 |
| **11Gb** | 234578 | 205094362 | 27380 | 874.31 | 501 | 1427 |
| **Table S5h. Statistic of each *de novo* assembly (stem)** | | | | | |  |
|  | **Total Sequences** | **Total bases** | **Max length** | **Average length** | **Median length** | **N50** |
| **1Gb** | 83195 | 59289411 | 15446 | 712.66 | 485 | 978 |
| **2Gb** | 111987 | 91060541 | 15216 | 813.13 | 526 | 1205 |
| **3Gb** | 133162 | 114443763 | 16997 | 859.43 | 538 | 1314 |
| **4Gb** | 150765 | 133154033 | 20954 | 883.19 | 544 | 1379 |
| **5Gb** | 166971 | 149963765 | 22973 | 898.14 | 546 | 1420 |
| **6Gb** | 181594 | 164221863 | 24425 | 904.34 | 547 | 1439 |
| **7Gb** | 194543 | 177516828 | 24465 | 912.48 | 549 | 1458 |
| **8Gb** | 206712 | 189641346 | 24486 | 917.42 | 548 | 1472 |
| **9Gb** | 218357 | 200892198 | 24507 | 920.02 | 550 | 1476 |
| **10Gb** | 228302 | 210197539 | 24507 | 920.7 | 552 | 1473 |
| **11Gb** | 238083 | 220493184 | 24507 | 926.12 | 555 | 1478 |

| **Table S6a. Length distribution of each *de novo* (apical bud)** | | | | | | |
| --- | --- | --- | --- | --- | --- | --- |
|  | **200-500** | **500-1000** | **1000-1500** | **1500-2000** | **2000-3000** | **>3000** |
| **1Gb** | 43105 | 22210 | 8303 | 3768 | 2440 | 748 |
| **2Gb** | 52991 | 28559 | 12579 | 6490 | 5054 | 2153 |
| **3Gb** | 61616 | 32479 | 15306 | 8436 | 6998 | 3466 |
| **4Gb** | 68926 | 36444 | 17094 | 9486 | 8292 | 4540 |
| **5Gb** | 76265 | 39482 | 18453 | 10672 | 9498 | 5400 |
| **6Gb** | 82936 | 42626 | 19589 | 11559 | 10415 | 6369 |
| **7Gb** | 88913 | 45241 | 21135 | 12105 | 11403 | 7134 |
| **8Gb** | 94348 | 48335 | 21866 | 12800 | 12045 | 8035 |
| **9Gb** | 99598 | 50722 | 23041 | 13416 | 12661 | 8664 |
| **10Gb** | 103672 | 53498 | 24067 | 14203 | 13147 | 9223 |
| **11Gb** | 107553 | 55957 | 24860 | 14731 | 13846 | 9730 |
| **Table S6b. Length distribution of each *de novo* (flower)** | | | | | | |
|  | **200-500** | **500-1000** | **1000-1500** | **1500-2000** | **2000-3000** | **>3000** |
| **1Gb** | 41879 | 18357 | 6754 | 3224 | 2214 | 865 |
| **2Gb** | 53246 | 24013 | 10129 | 5349 | 4310 | 2070 |
| **3Gb** | 63185 | 28440 | 12030 | 6742 | 5708 | 3193 |
| **4Gb** | 71383 | 32253 | 14060 | 7692 | 6807 | 4120 |
| **5Gb** | 78820 | 35369 | 15334 | 8678 | 7820 | 4988 |
| **6Gb** | 85288 | 38254 | 16672 | 9378 | 8837 | 5507 |
| **7Gb** | 91015 | 41763 | 17853 | 10040 | 9239 | 6291 |
| **8Gb** | 96635 | 44282 | 18934 | 10841 | 10013 | 6901 |
| **9Gb** | 101434 | 47175 | 19838 | 11548 | 10692 | 7311 |
| **10Gb** | 105910 | 49325 | 21222 | 11830 | 11265 | 7935 |
| **11Gb** | 110276 | 52139 | 21885 | 12332 | 11864 | 8453 |
| **Table S6c. Length distribution of each *de novo* (fruit)** | | | | |  |  |
|  | **200-500** | **500-1000** | **1000-1500** | **1500-2000** | **2000-3000** | **>3000** |
| **1Gb** | 43720 | 21372 | 7926 | 3817 | 2586 | 1015 |
| **2Gb** | 56365 | 28876 | 11700 | 6238 | 4987 | 2602 |
| **3Gb** | 65755 | 33793 | 14652 | 7783 | 6675 | 4002 |
| **4Gb** | 73799 | 38069 | 16563 | 9169 | 8001 | 5312 |
| **5Gb** | 80949 | 41983 | 18369 | 10252 | 9169 | 6305 |
| **6Gb** | 87315 | 45559 | 19870 | 11011 | 10121 | 7264 |
| **7Gb** | 92982 | 48438 | 21128 | 11614 | 11238 | 7982 |
| **8Gb** | 98154 | 51717 | 22278 | 12526 | 11813 | 8851 |
| **9Gb** | 102366 | 54740 | 23538 | 13039 | 12604 | 9483 |
| **10Gb** | 106793 | 57202 | 24816 | 13558 | 13142 | 10102 |
| **11Gb** | 110793 | 60098 | 25578 | 13947 | 13809 | 10753 |
| **Table S6d. Length distribution of each *de novo* (second young leaf)** | | | | | |  |
|  | **200-500** | **500-1000** | **1000-1500** | **1500-2000** | **2000-3000** | **>3000** |
| **1Gb** | 39239 | 21114 | 8479 | 3958 | 2713 | 1062 |
| **2Gb** | 49187 | 26903 | 12403 | 6674 | 5340 | 2604 |
| **3Gb** | 57940 | 31329 | 14894 | 8211 | 7223 | 3959 |
| **4Gb** | 65927 | 35462 | 16393 | 9368 | 8642 | 5148 |
| **5Gb** | 73158 | 39059 | 18331 | 10601 | 9651 | 6217 |
| **6Gb** | 79595 | 42752 | 19635 | 11312 | 10616 | 7144 |
| **7Gb** | 85806 | 45966 | 20763 | 12334 | 11456 | 7698 |
| **8Gb** | 91722 | 48743 | 22274 | 12854 | 12139 | 8473 |
| **9Gb** | 96885 | 51722 | 23250 | 13408 | 12890 | 9154 |
| **10Gb** | 101449 | 54766 | 24407 | 14042 | 13497 | 9713 |
| **11Gb** | 104959 | 56657 | 25116 | 14511 | 13846 | 10182 |
| **Table S6e. Length distribution of each *de novo* (mature leaf in summer)** | | | | | |  |
|  | **200-500** | **500-1000** | **1000-1500** | **1500-2000** | **2000-3000** | **>3000** |
| **1Gb** | 37021 | 18500 | 7354 | 3507 | 2480 | 961 |
| **2Gb** | 49621 | 25314 | 11181 | 5731 | 4869 | 2387 |
| **3Gb** | 59656 | 30294 | 13811 | 7411 | 6444 | 3581 |
| **4Gb** | 68485 | 34884 | 15634 | 8804 | 7831 | 4738 |
| **5Gb** | 75904 | 38590 | 17587 | 9689 | 9120 | 5742 |
| **6Gb** | 82527 | 42484 | 18954 | 10634 | 9974 | 6602 |
| **7Gb** | 88536 | 46065 | 20520 | 11464 | 10900 | 7297 |
| **8Gb** | 93843 | 49381 | 21698 | 12260 | 11444 | 8092 |
| **9Gb** | 98996 | 52582 | 22853 | 12905 | 12559 | 8553 |
| **10Gb** | 103596 | 55469 | 24138 | 13469 | 12953 | 9260 |
| **11Gb** | 108004 | 57688 | 25508 | 14253 | 13292 | 9757 |
| **Table S6f. Length distribution of each *de novo* (first young leaf)** | | | | | | |
|  | **200-500** | **500-1000** | **1000-1500** | **1500-2000** | **2000-3000** | **>3000** |
| **1Gb** | 37449 | 20688 | 8200 | 3929 | 2576 | 817 |
| **2Gb** | 45693 | 26380 | 12360 | 6525 | 5061 | 2214 |
| **3Gb** | 53485 | 30236 | 14470 | 8170 | 6901 | 3600 |
| **4Gb** | 60419 | 33719 | 16213 | 9603 | 8222 | 4541 |
| **5Gb** | 66365 | 36816 | 17679 | 10587 | 9278 | 5529 |
| **6Gb** | 71771 | 39771 | 18982 | 11431 | 10417 | 6393 |
| **7Gb** | 76955 | 42148 | 20255 | 12038 | 11370 | 7182 |
| **8Gb** | 82331 | 44551 | 21378 | 12895 | 11914 | 7769 |
| **9Gb** | 86622 | 47366 | 22134 | 13344 | 12685 | 8618 |
| **10Gb** | 90759 | 49751 | 23333 | 13694 | 13322 | 8957 |
| **11Gb** | 94383 | 52299 | 23997 | 14140 | 13947 | 9504 |
| **Table S6g. Length distribution of each *de novo* (root)** | | | | |  |  |
|  | **200-500** | **500-1000** | **1000-1500** | **1500-2000** | **2000-3000** | **>3000** |
| **1Gb** | 46609 | 20759 | 7476 | 3387 | 2166 | 831 |
| **2Gb** | 58204 | 28290 | 11689 | 5893 | 4402 | 2170 |
| **3Gb** | 66861 | 32685 | 14217 | 7538 | 6168 | 3451 |
| **4Gb** | 74826 | 36562 | 16230 | 8838 | 7443 | 4554 |
| **5Gb** | 81948 | 40029 | 17905 | 9838 | 8360 | 5628 |
| **6Gb** | 88675 | 43088 | 19095 | 10689 | 9533 | 6483 |
| **7Gb** | 94793 | 46266 | 20257 | 11419 | 10387 | 7192 |
| **8Gb** | 101059 | 48864 | 21472 | 12325 | 11036 | 7700 |
| **9Gb** | 106501 | 51803 | 22376 | 12728 | 12005 | 8264 |
| **10Gb** | 111808 | 54209 | 23407 | 13281 | 12527 | 8820 |
| **11Gb** | 116989 | 56423 | 24468 | 13830 | 13218 | 9650 |
| **Table S6h. Length distribution of each *de novo* (stem)** | | | | |  |  |
|  | **200-500** | **500-1000** | **1000-1500** | **1500-2000** | **2000-3000** | **>3000** |
| **1Gb** | 42801 | 23312 | 8873 | 4363 | 2784 | 1062 |
| **2Gb** | 53538 | 29779 | 13305 | 7021 | 5647 | 2697 |
| **3Gb** | 62482 | 34181 | 16108 | 8777 | 7428 | 4186 |
| **4Gb** | 70161 | 37972 | 17977 | 10368 | 9002 | 5285 |
| **5Gb** | 77537 | 41989 | 19564 | 11500 | 9984 | 6397 |
| **6Gb** | 84153 | 45703 | 20923 | 12462 | 11069 | 7284 |
| **7Gb** | 89672 | 49318 | 22301 | 13073 | 12041 | 8138 |
| **8Gb** | 95576 | 52128 | 23568 | 13711 | 12737 | 8992 |
| **9Gb** | 100352 | 55678 | 24816 | 14444 | 13615 | 9452 |
| **10Gb** | 104744 | 58260 | 26091 | 14925 | 14337 | 9945 |
| **11Gb** | 108651 | 61298 | 27322 | 15402 | 14681 | 10729 |

| **Table S7a. BUSCO statistics of each *de novo* (apical bud)** | | | | | | | | | | | |
| --- | --- | --- | --- | --- | --- | --- | --- | --- | --- | --- | --- |
|  | | **C** | | **S** | | **D** | | | **F** | | **M** |
| **1Gb** | | 32.50% | | 15.60% | | 16.90% | | | 23.70% | | 43.80% |
| **2Gb** | | 52.70% | | 23.30% | | 29.40% | | | 21.10% | | 26.20% |
| **3Gb** | | 64.10% | | 23.50% | | 40.60% | | | 15.40% | | 20.50% |
| **4Gb** | | 69.00% | | 22.40% | | 46.60% | | | 13.60% | | 17.40% |
| **5Gb** | | 73.50% | | 21.70% | | 51.80% | | | 11.00% | | 15.50% |
| **6Gb** | | 76.40% | | 21.20% | | 55.20% | | | 10.30% | | 13.30% |
| **7Gb** | | 78.10% | | 21.40% | | 56.70% | | | 9.90% | | 12.00% |
| **8Gb** | | 81.10% | | 22.60% | | 58.50% | | | 8.10% | | 10.80% |
| **9Gb** | | 82.90% | | 22.30% | | 60.60% | | | 6.90% | | 10.20% |
| **10Gb** | | 84.50% | | 22.00% | | 62.50% | | | 6.80% | | 8.70% |
| **11Gb** | | 85.40% | | 21.00% | | 64.40% | | | 6.80% | | 7.80% |
| **Table S7b. BUSCO statistics of each *de novo* (flower)** | | | | | | | | | | |  |
|  | | **C** | | **S** | | **D** | | | **F** | | **M** |
| **1Gb** | | 24.50% | | 13.10% | | 11.40% | | | 16.90% | | 58.60% |
| **2Gb** | | 38.30% | | 16.50% | | 21.80% | | | 18.30% | | 43.40% |
| **3Gb** | | 47.30% | | 20.10% | | 27.20% | | | 16.80% | | 35.90% |
| **4Gb** | | 53.10% | | 20.00% | | 33.10% | | | 16.00% | | 30.90% |
| **5Gb** | | 57.40% | | 20.70% | | 36.70% | | | 14.70% | | 27.90% |
| **6Gb** | | 61.90% | | 21.50% | | 40.40% | | | 13.60% | | 24.50% |
| **7Gb** | | 64.30% | | 20.30% | | 44.00% | | | 12.80% | | 22.90% |
| **8Gb** | | 66.80% | | 21.00% | | 45.80% | | | 12.40% | | 20.80% |
| **9Gb** | | 69.10% | | 20.10% | | 49.00% | | | 11.90% | | 19.00% |
| **10Gb** | | 70.70% | | 19.20% | | 51.50% | | | 11.90% | | 17.40% |
| **11Gb** | | 72.50% | | 20.80% | | 51.70% | | | 10.60% | | 16.90% |
| **Table S7c. BUSCO statistics of each *de novo* (fruit)** | | | | | | | | | | |  |
|  | | **C** | | **S** | | **D** | | | **F** | | **M** |
| **1Gb** | | 31.10% | | 16.40% | | 14.70% | | | 20.00% | | 48.90% |
| **2Gb** | | 48.10% | | 22.10% | | 26.00% | | | 18.40% | | 33.50% |
| **3Gb** | | 58.40% | | 22.60% | | 35.80% | | | 15.10% | | 26.50% |
| **4Gb** | | 63.10% | | 21.20% | | 41.90% | | | 14.40% | | 22.50% |
| **5Gb** | | 68.10% | | 22.40% | | 45.70% | | | 11.60% | | 20.30% |
| **6Gb** | | 70.80% | | 20.50% | | 50.30% | | | 10.60% | | 18.60% |
| **7Gb** | | 73.60% | | 20.80% | | 52.80% | | | 10.00% | | 16.40% |
| **8Gb** | | 74.40% | | 21.20% | | 53.20% | | | 9.70% | | 15.90% |
| **9Gb** | | 76.30% | | 21.20% | | 55.10% | | | 8.50% | | 15.20% |
| **10Gb** | | 78.40% | | 22.40% | | 56.00% | | | 8.10% | | 13.50% |
| **11Gb** | | 79.10% | | 21.50% | | 57.60% | | | 8.30% | | 12.60% |
| **Table S7d. BUSCO statistics of each *de novo* (second young leaf)** | | | | | | | | | |  |  |
|  | **C** | | **S** | | **D** | | **F** | **M** | |  |  |
| **1Gb** | 45.50% | | 21.70% | | 23.80% | | 17.60% | 36.90% | |  |  |
| **2Gb** | 63.00% | | 24.60% | | 38.40% | | 13.70% | 23.30% | |  |  |
| **3Gb** | 70.00% | | 21.50% | | 48.50% | | 10.80% | 19.20% | |  |  |
| **4Gb** | 72.90% | | 21.80% | | 51.10% | | 10.30% | 16.80% | |  |  |
| **5Gb** | 76.80% | | 21.00% | | 55.80% | | 8.70% | 14.50% | |  |  |
| **6Gb** | 78.90% | | 20.10% | | 58.80% | | 7.40% | 13.70% | |  |  |
| **7Gb** | 80.80% | | 19.60% | | 61.20% | | 7.00% | 12.20% | |  |  |
| **8Gb** | 81.70% | | 18.00% | | 63.70% | | 6.60% | 11.70% | |  |  |
| **9Gb** | 83.60% | | 18.00% | | 65.60% | | 5.70% | 10.70% | |  |  |
| **10Gb** | 84.10% | | 17.60% | | 66.50% | | 5.30% | 10.60% | |  |  |
| **11Gb** | 84.90% | | 17.40% | | 67.50% | | 4.90% | 10.20% | |  |  |
| **Table S7e. BUSCO statistics of each *de novo* (mature leaf in summer)** | | | | | | | | | |  |  |
|  | **C** | | **S** | | **D** | | **F** | **M** | |  |  |
| **1Gb** | 36.10% | | 15.60% | | 20.50% | | 19.30% | 44.60% | |  |  |
| **2Gb** | 53.60% | | 20.50% | | 33.10% | | 14.30% | 32.10% | |  |  |
| **3Gb** | 60.70% | | 20.10% | | 40.60% | | 13.80% | 25.50% | |  |  |
| **4Gb** | 66.40% | | 19.10% | | 47.30% | | 12.80% | 20.80% | |  |  |
| **5Gb** | 70.30% | | 20.10% | | 50.20% | | 11.50% | 18.20% | |  |  |
| **6Gb** | 73.50% | | 20.30% | | 53.20% | | 10.10% | 16.40% | |  |  |
| **7Gb** | 74.70% | | 19.00% | | 55.70% | | 9.40% | 15.90% | |  |  |
| **8Gb** | 77.10% | | 18.40% | | 58.70% | | 8.60% | 14.30% | |  |  |
| **9Gb** | 78.30% | | 18.00% | | 60.30% | | 8.30% | 13.40% | |  |  |
| **10Gb** | 78.60% | | 16.90% | | 61.70% | | 8.30% | 13.10% | |  |  |
| **11Gb** | 80.60% | | 18.40% | | 62.20% | | 7.40% | 12.00% | |  |  |
| **Table S7f. BUSCO statistics of each *de novo* (first young leaf)** | | | | | | | |  | |  |  |
|  | **C** | | **S** | | **D** | | **F** | **M** | |  |  |
| **1Gb** | 37.30% | | 18.30% | | 19.00% | | 22.20% | 40.50% | |  |  |
| **2Gb** | 55.90% | | 21.00% | | 34.90% | | 18.60% | 25.50% | |  |  |
| **3Gb** | 64.50% | | 21.00% | | 43.50% | | 14.90% | 20.60% | |  |  |
| **4Gb** | 70.00% | | 20.80% | | 49.20% | | 13.20% | 16.80% | |  |  |
| **5Gb** | 74.70% | | 22.50% | | 52.20% | | 10.70% | 14.60% | |  |  |
| **6Gb** | 79.10% | | 23.50% | | 55.60% | | 7.80% | 13.10% | |  |  |
| **7Gb** | 80.80% | | 22.50% | | 58.30% | | 7.00% | 12.20% | |  |  |
| **8Gb** | 81.90% | | 21.30% | | 60.60% | | 7.20% | 10.90% | |  |  |
| **9Gb** | 83.20% | | 21.20% | | 62.00% | | 6.90% | 9.90% | |  |  |
| **10Gb** | 84.80% | | 20.80% | | 64.00% | | 6.00% | 9.20% | |  |  |
| **11Gb** | 85.80% | | 19.70% | | 66.10% | | 5.60% | 8.60% | |  |  |
| **Table S7g. BUSCO statistics of each *de novo* (root)** | | | | | | | | | |  |  |
|  | **C** | | **S** | | **D** | | **F** | **M** | |  |  |
| **1Gb** | 23.10% | | 12.30% | | 10.80% | | 18.30% | 58.60% | |  |  |
| **2Gb** | 38.10% | | 18.20% | | 19.90% | | 18.20% | 43.70% | |  |  |
| **3Gb** | 47.70% | | 19.60% | | 28.10% | | 17.80% | 34.50% | |  |  |
| **4Gb** | 53.20% | | 20.60% | | 32.60% | | 17.40% | 29.40% | |  |  |
| **5Gb** | 58.70% | | 23.20% | | 35.50% | | 15.40% | 25.90% | |  |  |
| **6Gb** | 63.10% | | 22.10% | | 41.00% | | 14.00% | 22.90% | |  |  |
| **7Gb** | 65.10% | | 21.80% | | 43.30% | | 13.80% | 21.10% | |  |  |
| **8Gb** | 66.90% | | 20.80% | | 46.10% | | 13.40% | 19.70% | |  |  |
| **9Gb** | 69.30% | | 22.30% | | 47.00% | | 12.20% | 18.50% | |  |  |
| **10Gb** | 70.70% | | 20.10% | | 50.60% | | 12.20% | 17.10% | |  |  |
| **11Gb** | 72.80% | | 21.10% | | 51.70% | | 11.10% | 16.10% | |  |  |
| **Table S7h. BUSCO statistics of each *de novo* (stem)** | | | | | | |  |  | |  |  |
|  | **C** | | **S** | | **D** | | **F** | **M** | |  |  |
| **1Gb** | 33.80% | | 17.60% | | 16.20% | | 21.90% | 44.30% | |  |  |
| **2Gb** | 53.00% | | 23.30% | | 29.70% | | 19.20% | 27.80% | |  |  |
| **3Gb** | 63.70% | | 25.30% | | 38.40% | | 16.20% | 20.10% | |  |  |
| **4Gb** | 69.80% | | 23.60% | | 46.20% | | 13.40% | 16.80% | |  |  |
| **5Gb** | 73.30% | | 23.30% | | 50.00% | | 11.60% | 15.10% | |  |  |
| **6Gb** | 76.30% | | 21.60% | | 54.70% | | 10.10% | 13.60% | |  |  |
| **7Gb** | 78.10% | | 20.80% | | 57.30% | | 9.90% | 12.00% | |  |  |
| **8Gb** | 80.00% | | 20.80% | | 59.20% | | 8.30% | 11.70% | |  |  |
| **9Gb** | 81.30% | | 21.00% | | 60.30% | | 7.70% | 11.00% | |  |  |
| **10Gb** | 83.20% | | 21.30% | | 61.90% | | 7.30% | 9.50% | |  |  |
| **11Gb** | 83.60% | | 19.70% | | 63.90% | | 7.00% | 9.40% | |  |  |

| **Table S8a. Statistic of each *de novo* (apical bud and first young leaf)** | | | | | |  |
| --- | --- | --- | --- | --- | --- | --- |
|  | **Total Sequences** | **Total bases** | **Max length** | **Average length** | **Median length** | **N50** |
| **1Gb** | 103562 | 83181581 | 15964 | 803.21 | 526 | 1180 |
| **2Gb** | 139495 | 122552772 | 19586 | 878.55 | 544 | 1375 |
| **3Gb** | 167219 | 151091193 | 17586 | 903.55 | 543 | 1451 |
| **4Gb** | 190813 | 174641154 | 22392 | 915.25 | 545 | 1482 |
| **5Gb** | 210835 | 193853525 | 19132 | 919.46 | 545 | 1492 |
| **6Gb** | 228152 | 211659941 | 30383 | 927.71 | 547 | 1516 |
| **7Gb** | 243773 | 228215789 | 30491 | 936.18 | 550 | 1534 |
| **8Gb** | 258406 | 241972640 | 26158 | 936.4 | 552 | 1522 |
| **9Gb** | 270948 | 254960564 | 22310 | 940.99 | 556 | 1526 |
| **10Gb** | 282768 | 266621228 | 21966 | 942.9 | 557 | 1533 |
| **11Gb** | 292915 | 277525969 | 22515 | 947.46 | 563 | 1527 |
| **Table S8b. Statistics of each *de novo* (apical bud and root)** | | | | | | |
|  | **Total Sequences** | **Total bases** | **Max length** | **Average length** | **Median length** | **N50** |
| **1Gb** | 108700 | 85979580 | 17528 | 790.98 | 509 | 1164 |
| **2Gb** | 145648 | 127207249 | 18311 | 873.39 | 537 | 1367 |
| **3Gb** | 174710 | 156757724 | 24178 | 897.25 | 539 | 1438 |
| **4Gb** | 199364 | 181161910 | 24920 | 908.7 | 536 | 1478 |
| **5Gb** | 221206 | 202733777 | 24679 | 916.49 | 537 | 1495 |
| **6Gb** | 240290 | 221119281 | 24679 | 920.22 | 536 | 1511 |
| **7Gb** | 257550 | 237702272 | 20400 | 922.94 | 538 | 1511 |
| **8Gb** | 273157 | 252902768 | 33292 | 925.85 | 540 | 1517 |
| **9Gb** | 287551 | 267404051 | 30229 | 929.94 | 542 | 1522 |
| **10Gb** | 301091 | 280204740 | 35057 | 930.63 | 544 | 1517 |
| **11Gb** | 312880 | 292121071 | 26161 | 933.65 | 545 | 1531 |

| **Table S9a. Statistic of each *de novo* (apical bud, first young leaf and second young leaf)** | | | | | | |
| --- | --- | --- | --- | --- | --- | --- |
|  | **Total Sequences** | **Total bases** | **Max length** | **Average length** | **Median length** | **N50** |
| **1Gb** | 125986 | 108152091 | 19439 | 858.45 | 543 | 1312 |
| **2Gb** | 172428 | 156227618 | 22790 | 906.05 | 547 | 1446 |
| **3Gb** | 207310 | 191442574 | 23670 | 923.46 | 547 | 1496 |
| **4Gb** | 235997 | 219226666 | 21593 | 928.94 | 551 | 1505 |
| **5Gb** | 260610 | 243615189 | 19700 | 934.79 | 553 | 1515 |
| **6Gb** | 280952 | 265631501 | 25345 | 945.47 | 559 | 1536 |
| **7Gb** | 298572 | 283746095 | 20982 | 950.34 | 563.5 | 1535 |
| **8Gb** | 315059 | 301368832 | 25228 | 956.55 | 566 | 1545 |
| **9Gb** | 330390 | 316352144 | 25240 | 957.51 | 570 | 1540 |
| **10Gb** | 343519 | 330593096 | 26629 | 962.37 | 573 | 1553 |
| **11Gb** | 353762 | 340423464 | 27507 | 962.3 | 576 | 1544 |
| **Table S9b.Length distribution of each *de novo* (apical bud, first young leaf and second young leaf)** | | | | | | |
|  | **200-500** | **500-1000** | **1000-1500** | **1500-2000** | **2000-3000** | **>3000** |
| **1Gb** | 58647 | 32232 | 15646 | 8511 | 7260 | 3690 |
| **2Gb** | 79848 | 42528 | 20381 | 11775 | 11265 | 6631 |
| **3Gb** | 96007 | 51553 | 23500 | 13814 | 13139 | 9297 |
| **4Gb** | 108577 | 59292 | 26700 | 15429 | 15279 | 10720 |
| **5Gb** | 119207 | 66113 | 29374 | 17170 | 16659 | 12087 |
| **6Gb** | 127131 | 72404 | 31438 | 18013 | 18250 | 13716 |
| **7Gb** | 134293 | 77388 | 33909 | 19020 | 19134 | 14828 |
| **8Gb** | 140879 | 82375 | 35815 | 20046 | 19956 | 15988 |
| **9Gb** | 146750 | 86962 | 37936 | 20905 | 21002 | 16835 |
| **10Gb** | 152033 | 91206 | 38908 | 22049 | 21637 | 17686 |
| **11Gb** | 155704 | 94169 | 40601 | 22628 | 22617 | 18043 |
| **Table S9c.BUSCO of each *de novo* (apical bud, first young leaf and second young leaf).** | | | | | | |
|  | **C** | **S** | **D** | **F** | **M** |  |
| **1Gb** | 51.80% | 22.80% | 29.00% | 20.80% | 27.40% |  |
| **2Gb** | 70.20% | 24.40% | 45.80% | 12.10% | 17.70% |  |
| **3Gb** | 76.90% | 22.50% | 54.40% | 9.50% | 13.60% |  |
| **4Gb** | 80.40% | 20.30% | 60.10% | 7.60% | 12.00% |  |
| **5Gb** | 82.70% | 20.00% | 62.70% | 6.70% | 10.60% |  |
| **6Gb** | 85.40% | 19.70% | 65.70% | 5.80% | 8.80% |  |
| **7Gb** | 86.10% | 18.50% | 67.60% | 5.80% | 8.10% |  |
| **8Gb** | 87.90% | 18.00% | 69.90% | 4.90% | 7.20% |  |
| **9Gb** | 88.20% | 18.50% | 69.70% | 5.50% | 6.30% |  |
| **10Gb** | 88.40% | 17.40% | 71.00% | 5.20% | 6.40% |  |
| **11Gb** | 89.50% | 15.90% | 73.60% | 4.90% | 5.60% |  |

| **Table S10a. Runtime (hours) performance for each assembler at 0.5Gb** | | | | | |  |  |
| --- | --- | --- | --- | --- | --- | --- | --- |
|  | **K19** | **K21** | **K23** | **K25** | **K27** | **K29** | **K32** |
| **BinPacker** | 1.5 | 1.33 | 1.31 | 1.28 | 1.26 | 1.2 | 1.1 |
| **Bridger** | 1.66 | 1.67 | 1.65 | 1.66 | 1.65 | 1.67 | 1.66 |
| **SOAP***denovo* | 0.77 | 0.32 | 0.27 | 0.62 | 0.23 | 0.23 | 0.21 |
| **Trans-ABySS** | 0.098 | 0.099 | 0.083 | 0.087 | 0.087 | 0.081 | 0.11 |
| **Trinity** |  |  |  | 10.5 |  |  |  |
| **Table S10b. Runtime (hours) performance for each assembler at 1Gb** | | | | | |  |  |
|  | **K19** | **K21** | **K23** | **K25** | **K27** | **K29** | **K32** |
| **BinPacker** | 2.53 | 2.35 | 2.13 | 2.02 | 1.88 | 1.7 | 1.7 |
| **Bridger** | 4.75 | 3.33 | 2.6 | 2.6 | 2.63 | 2.6 | 2.68 |
| **SOAP***denovo* | 0.97 | 0.7 | 0.6 | 0.57 | 0.53 | 0.49 | 0.48 |
| **Trans-ABySS** | 0.15 | 0.142 | 0.154 | 0.142 | 0.143 | 0.142 | 0.183 |
| **Trinity** |  |  |  | 16.93 |  |  |  |
| **Table S10c. Runtime (hours) performance for each assembler at 3Gb** | | | | | |  |  |
|  | **K19** | **K21** | **K23** | **K25** | **K27** | **K29** | **K32** |
| **BinPacker** | 9.77 | 5.45 | 4.15 | 4.07 | 4.02 | 3.79 | 3.71 |
| **Bridger** | 5.66 | 4.57 | 4.36 | 3.98 | 4.14 | 4.13 | 4.09 |
| **SOAP***denovo* | 3.87 | 2.77 | 2.15 | 2 | 1.83 | 1.67 | 1.66 |
| **Trans-ABySS** | 0.31 | 0.33 | 0.3 | 0.32 | 0.3 | 0.32 | 0.44 |
| **Trinity** |  |  |  | 31.83 |  |  |  |

**Table S11. Completeness of transcriptomes generated in tea plant using PacBio technology.**

| **References** | **Complete** | **Complete and single-copy** | **Complete and duplicated** | **Fragmented** | **Missing** |
| --- | --- | --- | --- | --- | --- |
| Xu et al., 2017 | 55.1% | 35.6% | 19.4% | 11.6% | 33.3% |
| Qiao et al., 2019 | 92.7% | 38.6% | 54.1% | 3.2% | 4.1% |
